# Recent Advances and Developments in Injectable Conductive Polymer Gels for Bioelectronics

**DOI:** 10.1021/acsabm.3c01224

**Published:** 2024-02-16

**Authors:** Sergio J. Peñas-Núñez, David Mecerreyes, Miryam Criado-Gonzalez

**Affiliations:** † POLYMAT, University of the Basque Country UPV/EHU, Avda. Tolosa 72, 20018 Donostia-San Sebastián, Spain; ‡ Ikerbasque, Basque Foundation for Science, 48013 Bilbao, Spain

**Keywords:** conducting polymers, conductive nanomaterials, injectable gels, hybrid hydrogels, bioelectronics

## Abstract

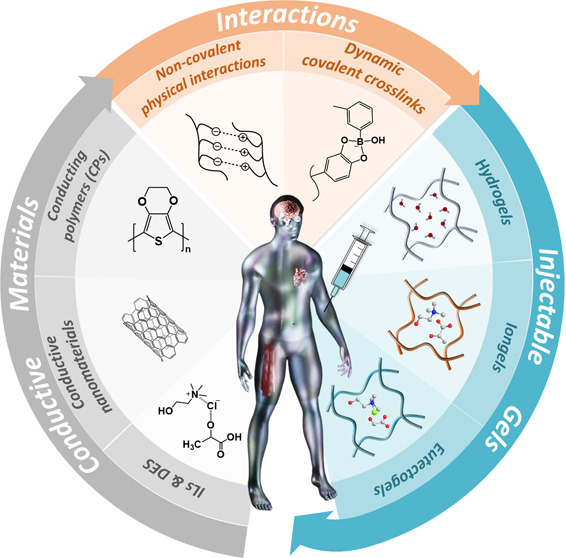

Soft matter bioelectronics represents an emerging and interdisciplinary research frontier aiming to harness the synergy between biology and electronics for advanced diagnostic and healthcare applications. In this context, a whole family of soft gels have been recently developed with self-healing ability and tunable biological mimetic features to act as a tissue-like space bridging the interface between the electronic device and dynamic biological fluids and body tissues. This review article provides a comprehensive overview of electroactive polymer gels, formed by noncovalent intermolecular interactions and dynamic covalent bonds, as injectable electroactive gels, covering their synthesis, characterization, and applications. First, hydrogels crafted from conducting polymers (poly(3,4-ethylene-dioxythiophene) (PEDOT), polyaniline (PANi), and polypyrrole (PPy))-based networks which are connected through physical interactions (e.g., hydrogen bonding, π–π stacking, hydrophobic interactions) or dynamic covalent bonds (e.g., imine bonds, Schiff-base, borate ester bonds) are addressed. Injectable hydrogels involving hybrid networks of polymers with conductive nanomaterials (i.e., graphene oxide, carbon nanotubes, metallic nanoparticles, etc.) are also discussed. Besides, it also delves into recent advancements in injectable ionic liquid-integrated gels (iongels) and deep eutectic solvent-integrated gels (eutectogels), which present promising avenues for future research. Finally, the current applications and future prospects of injectable electroactive polymer gels in cutting-edge bioelectronic applications ranging from tissue engineering to biosensing are outlined.

## 1 Introduction

Bioelectronics represents a dynamic and interdisciplinary research field that strategically harnesses the synergy between biology and electronics to advance diagnostic tools and healthcare treatments at the forefront of chemistry, engineering, physics, and materials science to pioneer innovative solutions.^1,2^ This is of paramount importance in the case of skin, muscle, tendon, nerve, and joint tissues, which respond and communicate via electrical signals and whose injuries cause human disability resulting in loss of mobility and sensory functions.^3,4^ Conventional electronics, relying on inorganic materials, face limitations in living-body-related applications, such as inherent stiffness and challenges in achieving long-term biocompatibility. In response, soft matter-based bioelectronics has emerged as a compelling alternative with the potential for addressing these drawbacks, which positions them as promising candidates for substantial growth in the coming years.^5,6^

**Scheme 1:**
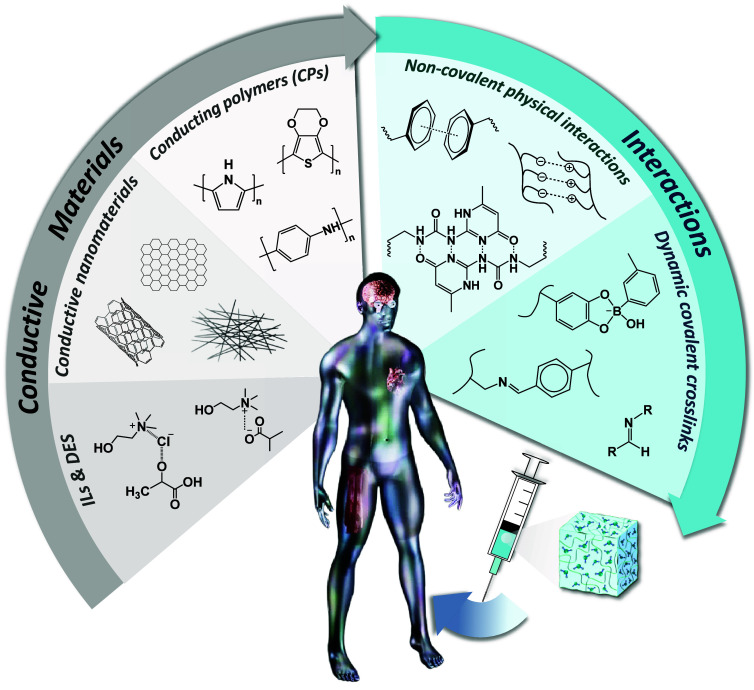
Schematic Illustration of the Electroactive Materials Employed for the Formation of Electroactive Injectable Gels through Physical Interactions and/or Dynamic Covalent Crosslinks to Be Employed in Bioelectronics

In this scenario, electroactive polymer hydrogels, which are formed by conducting polymer-based networks, or hybrid networks, which involve polymers and conductive nanomaterials, have the potential to act as a tissue-like space bridging the interface between the electronic device and dynamic biological fluids and body tissues.^7,8^ Although such conductive hydrogels can be wearable and implantable *in vivo* through surgical procedures, the current emphasis is based on advancing injectable hydrogels with self-healing properties. This strategic shift is motivated by their minimal invasiveness and their conformability to adapt to irregular body shapes. These properties make injectable hydrogels particularly promising for localized medical treatments.^9−11^ In Scheme 1, a comprehensive overview is presented, highlighting diverse materials utilized in the construction of injectable gels. These injectable gel networks predominantly encompass conducting polymers (CPs) (e.g., poly(3,4-ethylene-dioxythiophene) (PEDOT), polyaniline (PANi), and polypyrrole (PPy)), conductive nanomaterials (e.g., graphene oxide, carbon nanotubes, metallic nanoparticles, etc.), and ionic components (e.g., salts, ionic liquids (ILs), and deep eutectic solvents (DES)) linked through noncovalent physical interactions (e.g., hydrogen bonding, π–π stacking, hydrophobic interactions) or dynamic covalent bonds (e.g., imine bonds, Schiff bases, and borate ester bonds). Interestingly, when both organic electronic conductive materials and ionic conductive components are present within the gel network, organic mixed ionic/electronic conducting (OMIECs) gels are obtained.^12,13^ Moreover, owing to their injectability, they can be adeptly processed through extrusion-based 3D printing, enabling the creation of customized, shape-defined scaffolds for applications in bioelectronics.^14−16^ Injectable gels with electroactive properties have a variety of uses, either as carriers of therapeutic agents, like cells, drugs, and bioactive molecules, and/or as scaffolds for electrical stimulation of cells and tissues. This versatility makes them essential components in various bioelectronics applications, such as electronic skin (e-skin) and biosensors, among others.^17^

## 2 Synthesis and Characterization of Injectable Conductive Gels

Polymer gels, defined as three-dimensional (3D) cross-linked networks adept at retaining substantial volumes of biocompatible liquids, stand as prime candidates for interacting with biological fluids and tissues.^18,19^ Within this category, polymer gels formed by noncovalent intermolecular interactions and/or dynamic covalent bonds are garnering growing interest as injectable materials due to their self-healing ability and tunable biological mimetic features.^20^

Hydrogen bonding refers to dipole–dipole interactions between a hydrogen atom and an electronegative atom (i.e., O, N, or F). Polymers like polysaccharides and poly(vinyl alcohol) (PVA) possess abundant hydrogen-bond donors and acceptors, facilitating gelation through the formation of a significant number of hydrogen bonds. This gelation involves a competition with water molecules resulting in reversible cross-links. They are also thermosensitive because of the dissociation of the quadruple hydrogen bonding. In the case of electrostatic interactions, which commonly involve cationic polymers such as PEDOT interacting with anionic polymers and/or oppositely charged ions, weak ionic bonds with high chain mobility serve as reversible bonds to dissipate energy upon deformation, conferring injectability properties. π–π stacking is a strong physical interaction between aromatic rings via a plane-to-plane or an edge-to-plane orientation, which is also pH-sensitive. In all cases, the most interesting mechanism contributing to the injectability of physically cross-linked hydrogels is the bonds’ ability to break after stretching, transitioning from a sol-like state to a liquid-like state. Interestingly, owing to the dynamic nature of these physical interactions, the hydrogels can revert to the sol-like state after the applied strain is removed.^21^ The imine bond is a dynamic chemical cross-link obtained through condensation reactions of aldehydes and primary amines. Also known as a Schiff base, it shows pH-responsiveness and a reversible behavior during hydrolysis/reformation and imine exchange reactions to form injectable hydrogels. Another kind of dynamic covalent bond employed in the fabrication of injectable hydrogels is the boronic ester bond, which is formed via condensation reactions between boronic acids and 1,2- or 1,3-diols. They usually show strain-stiffening properties, which greatly enhance the polymer chain mobility and the reformation of the boronic acid–diol complexes via hydrolysis/reformation, leading to pH- and sugar-responsive injectable hydrogels.^22^

Injectable gels must have appropriate rheological behavior to be extruded, which is related to viscous or plastic behavior, and support a mass indefinitely without continued distortion, which is related to a solid-like behavior. During the injection process, the gel is subjected to stress (σ) and acquires strain (γ) at a rate of γ̇ as it is pushed through the needle. To that aim, polymer gels must possess a shear-thinning behavior exhibiting a decrease in viscosity with the share rate, thus allowing their extrusion. The yield stress (σ_γ_) is another critical parameter defined as the critical stress above which the gel will flow. In addition, polymer gels must have a self-healing behavior to achieve a fast and almost complete recovery of the solid-like state after injection.^23^ It is noteworthy that the ratio between the storage modulus (*G*′) and the loss modulus (*G*″), expressed as tan δ = *G*′/*G*″, also influences the injectability properties. An excessively high tan δ imparts a fluid-like behavior (*G*″ > *G*′) to the gel and favors the recovery of the solid-like state (*G*′ > *G*″) after injection. On the contrary, excessively low tan δ values may hinder the material’s ability to recover the gel state after injection.^24^ The incorporation of conductive polymers (CPs) into injectable gels allows the combination of viscoelastic properties with electronic conductivity. This development is particularly significant for the design of artificial bioelectronic scaffolds and devices, where precise control of mechanical and electrical signals is essential.^25,26^ This unique combination enables precise localization and external control of electrical stimuli directly at the target site.^27,28^

**Scheme 2:**
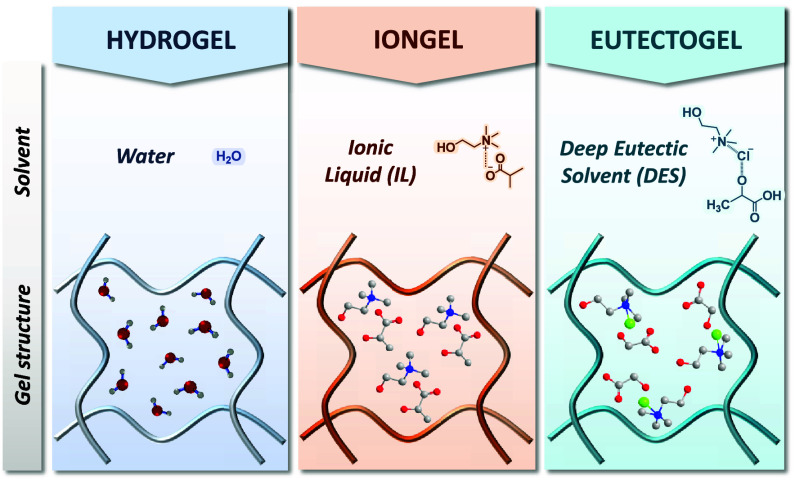
Schematic Representation of the Three Types of Electroactive Polymer Gel Networks Employed in Bioelectronics, Hydrogels, Iongels, and Eutectogels, Specifying the Kind of Solvent/Swelling Agent Employed in Each Case

Based on the choice of solvent or swelling agent, three distinct categories of injectable electroactive polymer gels can be discerned (Scheme 2): (i) Hydrogels, which employ water or a biologically relevant aqueous buffer (i.e., phosphate buffer solution (PBS), N-2-hydroxyethylpiperazine-N-2-ethanesulfonic acid (HEPES), etc.) as the swelling agent; (ii) Iongels, if the swelling agent is an ionic liquid (IL) (e.g., cholinium lactate, cholinium glycolate); and (iii) Eutectogels, characterized by the use of a deep eutectic solvent (DES) (i.e., lactic acid/choline chloride, glycerol/choline chloride, etc.) as the swelling agent.^13,29,30^

### 2.1 Injectable Conductive Hydrogels

#### 2.1.1 Electronically Conductive Polymers-based Hydrogels

Conducting polymers, long used in electronics, are becoming increasingly important in bioelectronics due to their inherent electronic conductivity, thermal and electrochemical stability, and biocompatibility. Among them, PEDOT, PANi, and PPy stand out as the most widely used CPs in the development of injectable hydrogels for bioelectronics.^31^

##### 2.1.1.1 PEDOT-based Injectable Hydrogels

**Figure 1:**
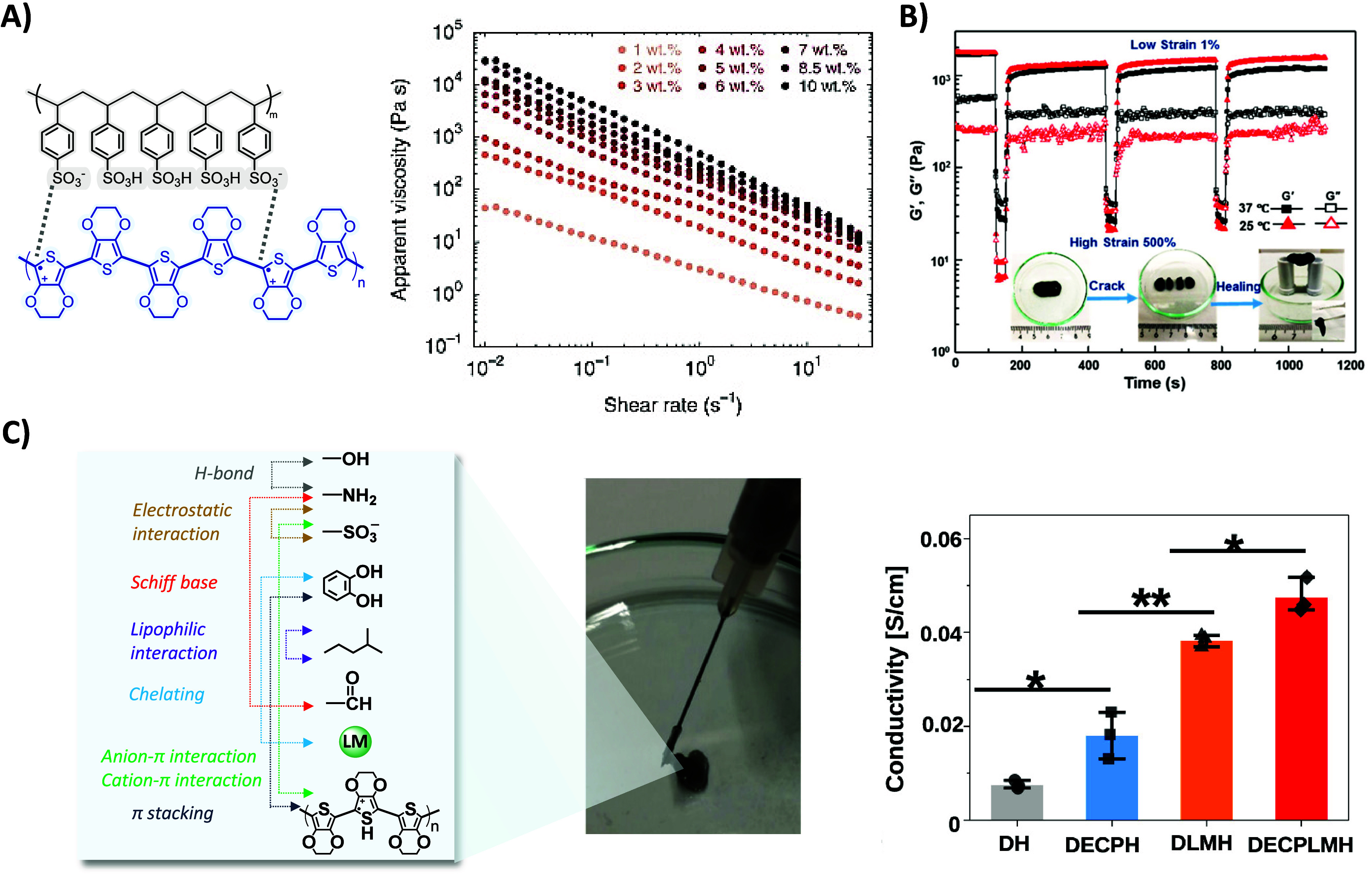
A) Schematic representation of the electrostatic interactions of PEDOT:PSS dispersions. Apparent viscosity as a function of shear rate of supramolecular PEDOT:PSS hydrogels by electrostatic interactions with DMSO of varying PEDOT:PSS nanofibril concentration. Reproduced with permission from reference (29). Copyright 2020 Springer Nature. B) Rheological dynamic step strain tests of supramolecular PEG-peptide/PEDOT:PSS hydrogels from 1 to 500% under different temperatures (25 and 37 °C). Insets are images showing the self-healing of the hydrogel. The hydrogel was cut into four pieces, put together, and healed into one block. Reproduced/adapted with permission from reference (37). Copyright 2018 American Chemical Society. C) Schematic representation of multiple interactions that contribute to forming dynamic electroconductive biopolymer/liquid metal hydrogels. Picture of the injected hydrogel via a syringe with a 27-gauge needle. Conductivity of the hydrogels, including Schiff-base cross-linked dynamic hydrogel (DH), dynamic electroconductive polymer hydrogel (DECPH), dynamic liquid metal hydrogel (DLMH), and dynamic electroconductive biopolymer/liquid metal hydrogels (DECPLMH). Reproduced with permission from reference (39). Copyright 2021 Springer Nature.

PEDOT is one of the most successful CPs from both a fundamental and a practical point of view, with a large number of applications in the field of bioelectronics. However, its insolubility and infusibility hinder its processability.^32^ To address this, PEDOT’s properties can be fine-tuned through the incorporation of counterions and secondary dopants.^33^ Commonly, PEDOT is doped with poly(styrene sulfonate) (PSS), resulting in stable aqueous diluted dispersions of PEDOT:PSS nanofibrils. However, the hydrogel formation is hindered by the low viscosity of PEDOT:PSS dispersions (∼10^1^ Pa·s). To obtain injectable hydrogels, Zhao and co-workers employed a cryogenic freeze-drying process of an aqueous PEDOT:PSS solution, followed by controlled redispersion in water/dimethyl sulfoxide (DMSO) (H_2_O:DMSO = 85:15% v/v). The viscosity of the suspensions gradually increased with the concentration of PEDOT:PSS nanofibrils (>10^2^ Pa·s), transitioning from a liquid-like to a gel-like state. The resulting hydrogel, formed through reversible physical entanglements among PEDOT:PSS nanofibrils and the solvent, exhibited characteristic shear-thinning and shear-yielding properties crucial for injectability applications, as confirmed by rheology (Figure 1A). Notably, the viscosity of the hydrogel played a pivotal role in injectability, as excessively high viscosities (>10^3^ Pa·s) risked clogging the needle nozzle. The injectability and self-healing attributes of these hydrogels enabled their processing through 3D printing techniques to produce well-defined conducting polymer electronic circuits, whose electrical conductivity varied with the nozzle diameter. Smaller nozzle diameters led to higher electrical conductivities (∼20 S cm^–1^) of the extruded hydrogels due to the shear-induced alignment of PEDOT:PSS nanofibrils (Young’s modulus = 1.1 MPa). The resulting conducting polymer hydrogels presented biocompatibility and long-term stability in physiological wet environments, maintaining their structural integrity after storing for 6 months in PBS.^29^

A similar strategy was followed by Khademhosseini et al., who employed 4-dodecylbenzenesulfonic acid (DBSA) to induce the gelation of PEDOT:PSS, which decreased from 300 to 2 min with increasing DBSA concentration from 3% v/v to 7% v/v. PEDOT:PSS exhibited a core–shell structure in dispersion, where the hydrophobic and positively charged thiophene rings of PEDOT formed the core, surrounded by the hydrophilic and negatively charged PSS chains. The addition of acidic DBSA, beyond the critical micelle concentration (CMC), increased the ionic strength protonating the SO_3_^–^ groups of PSS, which simultaneously weakened the electrostatic interaction between PEDOT and PSS, causing PEDOT interchain interactions via π–π stacking and hydrophobic interactions. This resulted in the formation of supramolecular hydrogels with a Young’s modulus of approximately 1 kPa. The weak and reversible physical cross-linking of these hydrogels allowed for easy injection with a syringe into the target area, ideal for potential applications in minimally invasive therapeutics. From a functional standpoint, Young’s modulus of the hydrogels needed to align with that of biological tissues, falling within the range of 1 to 100 kPa. To achieve this, the PEDOT:PSS hydrogel was infiltrated with precursors of a secondary hydrogel made of polyacrylamide (PAAm) or poly(2-hydroxyethyl methacrylate) (pHEMA). The resulting hydrogels featured interpenetrated networks of robust and covalently bonded PAAm or pHEMA hydrogels within the physically cross-linked PEDOT:PSS hydrogel. These hybrid hydrogels exhibited tunable mechanical properties with Young’s modulus values of 10 and 100 kPa for PAAm/PEDOT:PSS and pHEMA/PEDOT:PSS hydrogels, respectively. Moreover, the PEDOT:PSS hydrogel displayed a moderate electronic conductivity (∼10^–1^ S cm^–1^), plus excellent biocompatibility and stability, opening avenues for the development of soft and self-healing hydrogel bioelectronic devices.^34^

Although conductive hydrogels ensure a tissue-like soft modulus, they usually present weak tissue adhesiveness, hindering their fixing on the target tissues. This limitation could be overcome through the promising mussel-inspired strategy that enhances self-adhesiveness and introduces additional functional properties to hydrogel bioelectronics, particularly through the use of polydopamine (DA)-based hydrogels via catechol chemistry. Inspired by the mussel chemistry and dynamic borate chemistry, biocompatible and self-adhesive hydrogels were developed using a 4 wt % borate cross-linked network of pendant catechol groups formed by carboxymethyl cellulose–dopamine (CMC-DA). This network was embedded within a polyacrylamide (PAAm) cross-linked matrix containing 2 wt % PEDOT:PSS. The pendant DA chains, crucial for tissue adhesion (adhesion strength = 65 kPa), were cross-linked with borate ions within the covalently cross-linked PAAm network, enhancing self-healing and stretchability properties. Additionally, the incorporation of PEDOT:PSS not only increased the elastic modulus, from ∼2.5 kPa to ∼3.1 kPa, and stretchability, from 2763% to 2873%, but also conferred conductivity (41.6 S m^–1^) through charge transfer between PEDOT and catechol/quinone groups of CMC-DA. This facilitated the formation of a well-connected electric path in the hydrogel, which also exhibited long-term adhesion and biocompatibility.^35^ Likewise, hydrogels based on catechol-conjugated alginate (Alg-CA) cross-linked with Ca^2+^ and incorporating PEDOT:PSS were developed. The elastic modulus (*G*′) exhibited a notable rise, from 10^2^ Pa for bare Alg-CA to 3 × 10^3^ Pa for Alg-CA/PEDOT:PSS due to the π–π stacking interactions between the ring structures of Alg-CA and PEDOT:PSS. These physically cross-linked hydrogels demonstrated adhesive and shear-thinning characteristics, evidenced by a decrease in viscosity with increasing shear rate. This property was advantageous for enhanced flow through narrow gauges. The hydrogels also displayed self-healing capabilities; the initial *G*′ of approximately 3 × 10^3^ Pa at low strains (0.5%) significantly decreased to ∼3 Pa after applying a high strain (1000%) but promptly recovered upon strain removal. Concurrently, the conductivity of the hydrogels increased with the concentration of PEDOT:PSS, rising from ∼2.5 × 10^–3^ to ∼4 × 10^–3^ S cm^–1^ for hydrogels containing 0.5% and 1% PEDOT:PSS, respectively. These conductivity values were comparable to those of muscle tissues, reinforcing the potential of these hydrogels in bioelectronic applications.^36^

There is a keen interest in integrating cells within injectable hydrogels to develop artificial biomimetic tissue for regenerative medicine purposes close to clinical applications. In this sense, Zhang and co-workers introduced a physically cross-linked hydrogel by combining polyethylene glycol-peptide (PEG-peptide) with PEDOT:PSS. Diverse CWGG(BX)_n_ peptides were synthesized, where B represented a basic residue (i.e., arginine or lysine) and X denoted alanine, glycine, or serine. These peptides were then linked to maleimide-functionalized PEG through Michael-type addition reactions, utilizing the thiol group of the cysteine amino acid in the peptide chain. When incorporated into PEG-peptide, PEDOT:PSS formed homogeneous supramolecular hydrogels through a combination of various physical interactions. These included electrostatic interactions between the positively charged peptide and the negatively charged PSS, π–π stacking among PEDOT nanoparticles, and anion−π interactions between PEDOT and PSS. To validate the shear-thinning and self-healing properties of the PEG-peptide/PEDOT:PSS hydrogels, continuous flow experiments and step-strain rheological studies were conducted. The viscosity exhibited a decrease with shear rate, indicative of the network rearrangement during high shear rate-induced flow, confirming the shear-thinning behavior. Regarding self-healing properties, Figure 1B illustrates that at low strains (1%), the elastic modulus (*G*′ ∼ 10^3^ Pa) surpassed the loss modulus (*G*″ ∼ 10^2^ Pa), indicating a gel-like state. After the application of a substantial strain (500%), it transitioned to a liquid-like state (*G*″ > *G*′). The gel-like state was rapidly restored upon strain removal (*G*′ > *G*″), although 20 min were required for the complete recovery of initial stiffness (*G*′) values. Notably, this combinatorial approach led to hydrogels with a denser molecular packing of PEDOT nanoparticles, resulting in enhanced conductivity. Cyclic voltammograms (CV) exhibited the typical oxidation peak of PEDOT between 0.5 and 0.7 V, with reduction occurring between −0.1 and −0.02 V, which promoted the proliferation and differentiation of encapsulated mesenchymal stromal cells upon electrical stimulation. The hydrogels were stable after incubation in PBS or cell culture medium over a period of six months.^37^ Spencer et al. developed biocompatible hydrogels, both *in vitro* and *in vivo*, made of gelatin methacryloyl (GelMA) and PEDOT:PSS that underwent a two-step formation process: initially, PEDOT:PSS was physically cross-linked with Ca^2+^ ions, imparting injectability properties, followed by the visible-light-induced photopolymerization cross-linking of the methacryloyl groups of GelMA. The conductivity of the hydrogels increased with the concentration of PEDOT:PSS, unaffected by the varying concentration of CaCl_2_. Additionally, the presence of PEDOT:PSS influenced the mechanical properties, with variations of the modulus from ∼141.6 kPa to ∼80.0 kPa as the PEDOT:PSS content increased from 0.1% and 0.3%. The hydrogels degraded *in vivo* with no substantial inflammatory responses.^38^

The high acidity of the residual sulfonate groups of PSS made the systems not fully biocompatible and hindered their biomedical applications in certain scenarios. To address this issue, alternative counterions such as polysaccharides and glycosaminoglycans (GAGs) have been explored. Mecerreyes and co-workers successfully used hyaluronic acid (HA), chondroitin sulfate (CS), and heparin (HEP), to synthesize PEDOT dispersions by chemical oxidative polymerization. The PEDOT:GAGs dispersions demonstrated enhanced support for neuroregenerative processes compared to commercial PEDOT:PSS, evidenced by the longer neurite lengths observed in differentiated SH-SY5Y cells.^40^ Dynamic electroconductive hydrogel networks with shear-thinning and self-healing properties were formed through different reversible bonds, including covalent imine bonds (Schiff bases) and noncovalent interactions (electrostatic interactions, H-bonds, hydrophobic interactions, π–π stacking, anion/cation−π interactions, and complexation liquid metal–polyphenol group) (Figure 1C). They were obtained by mixing tannic acid (TA)-coated liquid metal (LM) nanodroplets (LM-TA) with catechol-functionalized chitosan (CHI-CA), cholesteryl, aldehyde-modified dextran (Dex-ALD-CH), and PEDOT:HEP. These characteristics endowed the hydrogel with the capability to be extruded through a 27-gauge needle (inner diameter = 0.21 mm), a desirable property for surgical practice. These hydrogels exhibited electroactive properties with conductivity values around 5 × 10^–2^ S cm^–1^, and long-term cell proliferation remaining at the subcutaneous injection site for a long time.^39^

Li and co-workers employed a similar strategy to fabricate PEDOT-based hydrogels using dynamic covalent imine bonds. Initially, EDOT was polymerized in the presence of dextran sulfate (Dex), resulting in a PEDOT:Dex dispersion, which was freeze-dried and redispersed in an aqueous solution of carboxymethyl chitosan (CMCHI), yielding a PEDOT:(Dex/CMCHI) dispersion with conductivity values of ∼1.2 × 10^–2^ S cm^–1^ and enhanced biocompatibility than commercial PEDOT:PSS. The next step involved creating injectable hydrogels by simultaneously mixing the solutions of PEDOT:(Dex/CMCHI) and oxidized dextran (ODex) using two syringes. The formation of dynamic covalent imine bonds between PEDOT:(Dex/CMCHI) and ODex facilitated the gelation in 1 min due to the formation of electrostatic and π–π interactions, which aligns with the “time window” necessary for surgical applications. Furthermore, the Young’s modulus of the hydrogels could be adjusted by varying the PEDOT:Dex content in the range of 10–25 kPa, assimilating to the Young’s modulus typical of cardiac tissue. The hydrogels exhibited long-term stability for optimal implant longevity.^41^

Liu et al. reported a genetically targeted approach for orchestrating the assembly of conductive polymers within neurons. These innovative techniques induced a remodeling of membrane electrical properties, facilitating cell-type-specific cellular and behavioral modulation, as evidenced by the control of neuronal firing in cultures of rat hippocampal neurons, mouse brain slices, human cortical spheroids, and in living *Caenorhabditis elegans* worms, as well as long-term biocompatibility.^42^ In this line, Berggren and co-workers carried out the *in vivo* creation of electrodes in zebrafish and leech models, using endogenous metabolites to trigger enzymatic polymerization of organic precursors within an injectable gel, thereby yielding conducting polymer gels with long-range conductivity, which is particularly promising for nerve stimulation applications. They demonstrated the successful long-term neural stimulation and recording in a seamless manner.^43^

##### 2.1.1.2 PPy-based Injectable Hydrogels

**Figure 2:**
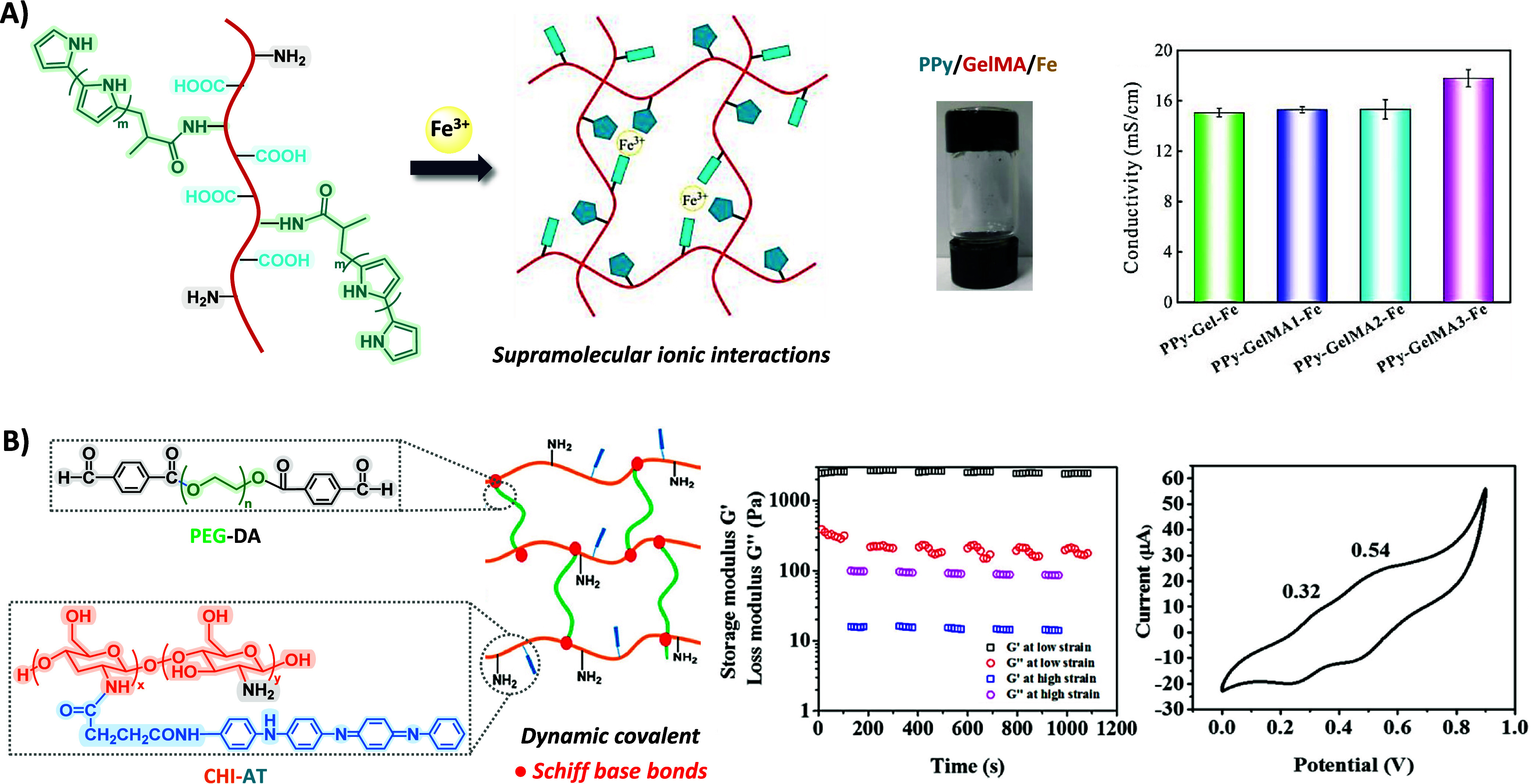
A) Schematic representation of the formation of supramolecular PPy-MAC-Fe hydrogels by ionic interactions and a representative picture of hydrogels formed. Conductivity values of the PPy-GelMA-Fe hydrogels. Reproduced/adapted with permission from reference (45). Copyright 2020 American Chemical Society. B) Schematic representation of injectable CHI-AT/PEG-DA hydrogels through dynamic covalent Shiff base bonds. Rheological properties of the hydrogel when alternate step strain switched from 1% to 300%. Cyclic voltammogram of the CHI-AT_20_ hydrogel doped in a mixture of DMSO and 1 M HCl. Reproduced/adapted with permission from reference (49). Copyright 2016 American Chemical Society.

Apart from PEDOT, polypyrrole (PPy) has also been used as an electroconductive component to develop injectable hydrogels. PPy was copolymerized with methacrylated collagen (MAC) using the redox initiator/oxidants (ammonium persulfate (APS), iron(III) tosylate, and N,N,N,N-tetramethylethylenediamine (TEMED)), as well as N,N′-methylenebis(acrylamide) (MBAA) as a cross-linker of the methacrylate groups in MAC, resulting in an interpenetrated biocoampatible hydrogel network PPy-MAC-Fe with conductivity values of 3.4 × 10^–3^ S cm^–1^. The mechanical properties (from 1 to 3 kPa) varied with the MAC concentration (from 20 wt % to 50 wt %).^44^ A similar approach was employed by Shen Wang et al. to obtain polypyrrole-grafted gelatin-based hydrogels. In this case, polypyrrole was grafted onto methacrylic-anhydride-modified gelatin (PPy-GelMA) by reacting with the double bond. The hydrogels were later formed through reversible ionic interactions of PPy-GelMA with ferric ions (Figure 2A), which endowed them with injectability properties. Notably, PPy-GelMA-Fe hydrogels possessed good conductivity (∼1.6 × 10^–2^ S cm^–1^), due to the presence of both polypyrrole and ferric ions, which surpassed those values of PPy-GelMA hydrogels lacking Fe and those composed of MAC-Fe without incorporating PPy. Additionally, the hydrogels demonstrated noncytotoxicity and compatibility with blood, further enhancing their appeal for biomedical applications.^45^

##### 2.1.1.3 PANi-based Injectable Hydrogels

Multifunctional injectable hydrogels that integrated conductivity, antioxidant, and antibacterial properties were obtained through Schiff base bonds between amino groups of N-carboxyethyl chitosan (CEC) and amino groups of oxidized hyaluronic acid-graft-aniline tetramer (OHA-AT) (molar ratio −CHO:–NH_2_ = 1:1) under physiological conditions (phosphate buffer solution (PBS), pH 7.4, 37 °C). The gelation time increased with the AT content, which compacted OHA-AT molecules in an aqueous solution due to their hydrophobic nature leading to a decrease of the chain entanglements among the OHA-AT macromolecules. The AT content also induced a decrease in *G*′ from 1400 to 900 Pa due to a decrease in the cross-linking density between CHI and OHA, as evidenced by an increase in pore size from 19.5 μm to 36.7 μμm, characterized by scanning electron microscopy (SEM). Cyclic voltammetry (CV) curves exhibited two pairs of reduction/oxidation peaks at 0.45 and 0.72 V corresponding to transitions between the leucoemeraldine state to the emeraldine state, and from the emeraldine oxidation state of AT to the pernigraniline state, respectively. Moreover, the conductivity of the hydrogels increased with AT content, from ∼5 × 10^–5^ S cm^–1^ for CEC/OHA to ∼4.2 × 10^–4^ S cm^–1^ for CEC/OHA-AT. The hydrogels showed *in vivo* biocompatibility, which increased with the AT content, and a linear-like degradation behavior under physiological mimicking conditions (PBS pH 7.4, 37 °C) with a 74% weight loss after 8 days.^46^ Saeb and co-workers developed injectable hydrogels formed via Schiff base bonds between −NH_2_ groups of CHI from chitosan (CHI)-graft-N-hydroxysuccinimide (NHS) capped aniline pentamer (AP) and −CHO groups of Pluronic F127, in which AP provided electroactivity and antibacterial properties, and Pluronic endowed them with injectability. The gelation time decreased with the Pluronic concentration because of an increase in the cross-linking degree, whereas *G*′ decreased with the percentage of AP as its steric hindrance disrupted the lower critical solution temperature (LCST) behavior of Pluronic. Rheological tests pointed out a liquid-like state (*G*′ < *G*″) at low temperatures, while a sol–gel transition (*G*″ > *G*′) was achieved by increasing the temperature. Ultimately, CHI-AP-Pluronic hydrogels were biocompatible and achieved a desirable conductivity (∼10^–3^ S cm^–1^) for on-demand electro-responsive drug release applications.^47^

A similar strategy was adopted to obtain injectable hydrogels made of quaternized chitosan-graft-polyaniline (CHI-PANi) and benzaldehyde-group-functionalized poly(ethylene glycol)-co-poly(glycerol sebacate) (PEG-PGS), which possessed a multifaceted set of properties, including conductivity (∼10^–3^ S cm^–1^) similar to skin components, biocompatibility, and antibacterial and antioxidant properties for wound healing. The gelation time decreased with PEG-PGS concentration (0.5 and 2 wt %) from 374 to 86 s due to the increase in the cross-linking density. Hydrogels with lower cross-linking density and *G*′ ∼ 60 Pa degraded after 33.5 h, whereas hydrogels with higher cross-linking density and *G*′ ∼ 370 Pa remained stable after swelling in PBS.^48^ On the other hand, CHI-AT/PEG-DA hydrogels exhibited higher elastic moduli (*G*′ ∼ 2 × 10^3^ to ∼1.2 × 10^4^ Pa). Rheological analysis using the step strain method demonstrated the hydrogels’ self-healing behavior, attributed to the dynamic covalent Schiff-base interactions between amine groups of CHI-AT and benzaldehyde groups of PEG-DA chain. Hydrogels exhibited two pairs of reduction/oxidation peaks in the CV curves owing to the transitions from “leucoemeraldine” to the “emeraldine” state (0.32 V) and from the “emeraldine” to the “pernigraniline” state (0.54 V), and conductivity values (∼10^–3^ S cm^–1^) that are quite close to native cardiac tissue (Figure 2B). *In vivo* tests showed the complete degradation of the hydrogels after 45 days.^49^ Building on this approach, the same researchers explored a parallel system employing dextran-graft-aniline tetramer-graft-4-formylbenzoic acid (Dex-AT-FA) and N-carboxyethyl chitosan (CEC) as integral components of biocompatible injectable hydrogels, which served as cell-laden bioinks, encapsulating both C2C12 myoblasts and human umbilical vein endothelial cells (HUVEC) for facilitating the repair of skeletal muscle.^50^ Another example reported the employment of hydrogen-bonding 2-ureido-4[1H]-pyrimidinone (UPy) groups as cross-linkers of a PANi/PSS network to form an interpenetrated network that conferred the hydrogel electronic conductivity assisted by ionic transport (∼1.3 × 10^–1^ S cm^–1^). Remarkably, the hydrogels exhibited rapid and complete self-healing within 30 s, which showcases their use as flexible wearable sensors.^51^

#### 2.1.2 Injectable Hybrid Electronic Conductive Hydrogels

Hybrid injectable hydrogels have been developed through the incorporation of conductive nanomaterials within polymer hydrogel networks.^52^

##### 2.1.2.1 Hybrid Polymeric Hydrogels from Conductive Carbonaceous Nanomaterials

Carbon nanotubes (CNTs) and graphene oxide (GO) have long been central to the development of conductive materials due to their large surface areas and superior electronic conductivity.^53,54^

Conductive, stretchable, and mussel-inspired adhesive hydrogels were formed by integrating nanosheets, formed by the self-assembly of PEDOT on a polydopamine-reduced and sulfonated graphene oxide (PSGO) template, into a polyacrylamide (PAAm) hydrogel network. The nanosheets not only imparted high conductivity to the hydrogel but also established physical cross-links within the chemically cross-linked PAAm network, contributing to its stretchability. They also presented *in vivo* biocompatibility and long-term adhesiveness.^55^ CNTs were seamlessly integrated into collagen (COL) hydrogels, yielding noncytotoxic hybrid COL/CNTs hydrogels with enhanced mechanical properties (*G*′ = 29 kPa) and a 3-fold increase in electronic conductivity (∼6 × 10^–3^ S cm^–1^).^53^ Hybrid injectable hydrogels were developed by Guo and co-workers through the incorporation of ultrasmall graphene quantum dots (GQDs) into CHI/COL hydrogel networks with good biocompatibility and biodegradability for cardiac therapies.^56^ In another example, injectable nanocomposite conductive hydrogels were obtained through the physical H-bonding and π–π stacking between hyaluronic-acid-graft-dopamine (HA-DA) and reduced graphene oxide (rGO). The gelation time decreased with the percentage of rGO, from 557 s for HA-DA to 158 s for HA-DA/rGO hydrogels, and the conductivity reached values of ∼5 × 10^–3^ S cm^–1^.^57^

**Figure 3:**
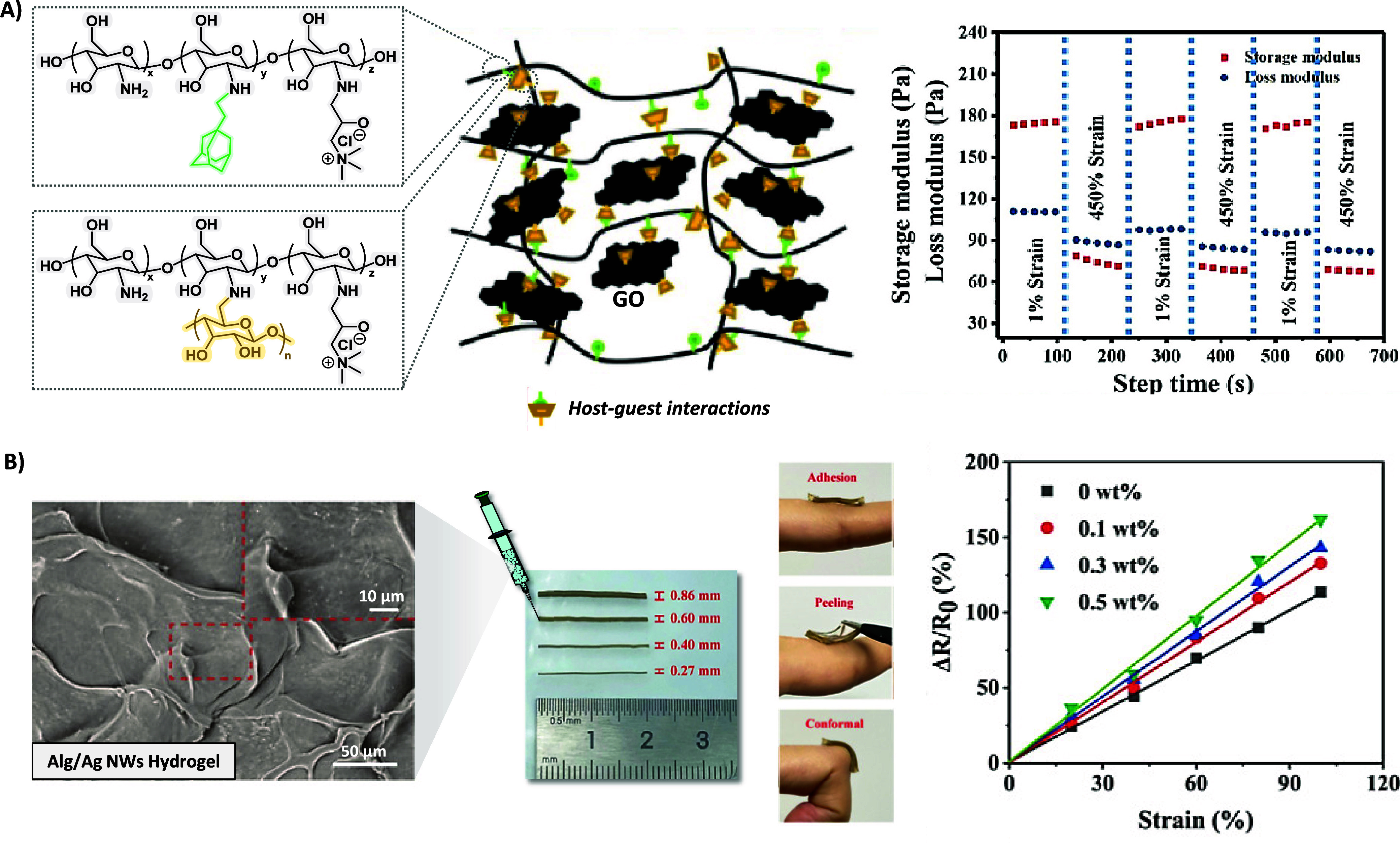
A) Schematic representation of the formation of supramolecular CHI-CD-AD/GO hydrogels through host–guest interactions. Rheological dynamic step strain tests of the CHI-CD-AD/GO hydrogels from 1% to 450%. Reproduced/adapted with permission from reference (58). Copyright 2020 Elsevier. B) Representative SEM picture of a hybrid Alg-PBA/Alg-DA hydrogel containing 0.5 wt % Ag NWs, hydrogel wires with different lengths and diameters after passing through different size needles, photos that demonstrate the conformal adhesion of the hydrogel, and relative resistance changes as a function of strain values. Reproduced/adapted with permission from reference (61). Copyright 2022 American Chemical Society.

Beilin Zhang et al. designed injectable conductive hydrogels through host–guest interactions of quaternized chitosan-graft-cyclodextrin (CHI-CD), quaternized chitosan-graft-adamantane (CHI-AD), and graphene oxide-graft-cyclodextrin (GO-CD) (Figure 3A), which possessed electrical conductivity (∼1.1 × 10^–3^ S cm^–1^) similar to that of the skin. The injectability properties of CHI-CD-AD/GO hydrogels were assessed by the rheological dynamic strain step test, in which the materials presented a reversible transition from a gel-like state at low strains to a liquid-like state at high strains.^58^ In another approach, biocompatible hybrid hydrogels were made of a sodium tetraborate cross-linked poly(vinyl alcohol) (PVA) matrix with embedded hydroxypropyl cellulose (HPC) short-chain fibers and CNTs. The self-healing capabilities were achieved by a ternary heterogeneous network of cross-linking and abundant dynamic H-bonds.^59^

##### 2.1.2.2 Hybrid Polymeric Hydrogels from Conductive Metallic Nanomaterials

Apart from carbonaceous nanomaterials, self-healable hybrid hydrogels were developed by dynamic borate ester bonds and H-bonds between Ag/MXene nanocomplexes and PVA chains. The conductivity of the hydrogel increased with the Ag/MXene content from 3 × 10^–4^ S cm^–1^ (0.1 wt %) to 2.6 × 10^–3^ S cm^–1^ (1 wt %), which could be attributed to the formation of more conductive pathways in the hydrogel network. In addition, Ag/MXene conferred antibacterial activity to the hydrogels.^60^ In a similar approach, silver nanowires (Ag NWs) were embedded into a self-healable hydrogel made of Alg modified with 3-aminophenylboronic acid (Alg-PBA) and Alg modified with dopamine (Alg-DA) (Figure 3B). The hydrogels were successfully extruded through syringes of different sizes, featuring tip diameters spanning from 0.86 mm to 0.27 mm. Notably, the introduction of Ag NWs induced morphological changes in the Alg hydrogels, transforming them from empty pore structures to Ag NWs-filled pore networks (Alg-PBA/Alg-DA/Ag NWs) as they formed a secondary conductive network within the hydrogel (∼2 × 10^–3^ S cm^–1^). This structural modification resulted in an increased Young’s modulus, escalating from ∼10 kPa for Alg hydrogels to approximately ∼15.6 kPa for Ag NWs-loaded hydrogels. According to the experimental findings, the hydrogels were reusable for at least 3 cycles, translating to an effective usage span of approximately 9 days.^61^ Beyond that, physically cross-linked hydrogels were obtained via multiple H-bonds between carboxymethyl cellulose (CMC) and tannic acid (TA), and metal–carboxyl and metal–phenol coordination due to the incorporation of metal ions (chloroauric acid (HAuCl_4_), silver nitrate (AgNO_3_), and ferric chloride (FeCl_3_)). While all hydrogels exhibited a reversible conductivity under cyclic strains from 0 to 400%, those containing Au ions possessed the highest conductivity whereas those based on Fe ions presented the lowest conductivity.^62^

#### 2.1.3 Ionic Conductive Injectable Hydrogels

While conducting polymers and electroactive carbonaceous and metallic nanomaterials provide electronic conductivity to the hydrogels, the presence of salts within the hydrogels network can endow them with ionic conductivity. Noncytotoxic ionic conductive injectable hydrogels were obtained by soaking hydrogels made of boronic-acid-modified alginate (Alg-BA) cross-linked with oligomerized epigallocatechin gallate (OEGCG) into a NaCl solution. The boronic-acid–*cis*-diol dynamic covalent bonds conferred stretchability (up to 500%) and self-healing properties, while the NaCl concentration, apart from provided ionic conductivity, also increased the elastic modulus from ∼3 × 10^3^ Pa for Alg-BA/OEGCG up to ∼1 × 10^4^ Pa for Alg-BA/OEGCG/NaCl. Resistance–strain cyclic tests demonstrate a resistance decrease with NaCl concentration, from 1.7 kΩ for Alg-BA/OEGCG up to 1 kΩ for Alg-BA/OEGCG/NaCl with 2% NaCl.^63^ Another example reported H-bonds cross-linked hydrogels of PAAm and carrageenan (CAR), in which NaCl and KCl solutions endowed hydrogels with ionic conductivity (∼3.8 × 10^–2^ S cm^–1^).^64^

Ghost and co-workers adopted a similar strategy to develop ionic conductive injectable hydrogels, which combined physical and dynamic covalent interactions between carboxymethyl chitosan (CMCHI) and polydopamine-conjugated carboxymethyl cellulose dialdehyde (CMC-DA), immersed in PBS. The ionic conductivity (∼3 × 10^–3^ S cm^–1^) was attributed to the metal coordination of ions present in PBS (e.g., Na^+^, Mg^2+^, and Ca^2+^) along with the carboxylic groups in CMCHI raising positive charge capacity and the charge delocalization.^65^ However, from the functional point of view, the water evaporation along the lifetime of the hydrogels in the bioelectronics application can lead to a subsequent loss of valuable mechanical and electrical properties.

### 2.2 Conductive Polymer Injectable Iongels or Ionic Liquid Gels

Ionic liquid integrated gels (iongels) emerged as ideal candidates to overcome the limitations of hydrogels such as loss of properties due to water evaporation. Ionic liquids (ILs) are formed by molten salts and possess nonvolatility properties, thermal stability, high ionic conductivity, and electrochemical stability, which complement the shortcomings of injectable polymer gels for bioelectronics and provide new functionalities.^64^ Injectable iongels are formed by physical and/or dynamic covalent cross-linked polymer networks in the presence of ILs, which act as solvent and swelling agents. For example, iongels were developed by supramolecular cross-linking between polyphenols (i.e., gallic acid, pyrogallol, and tannic acid) and PVA in the presence of different kinds of ILs, such as imidazolium-based ILs (1-ethyl-3-methylimidazolium dicyanamide and 1-ethyl-3-methylimidazolium bromide) and cholinium-based ILs (combining acetate or lactate anions).^66,67^ The resulting iongels exhibited flexibility and high ionic conductivity (∼1.8 × 10^–2^ S cm^–1^). In addition, ILs containing the cholinium cation and different phenolic acid anions (i.e., cholinium salicylate ([Ch][Sal]), cholinium gallate ([Ch][Ga]), cholinium vanillate ([Ch][Van]), and cholinium caffeate ([Ch][Caff])) demonstrated noncytotoxic properties and commendable anti-inflammatory, antioxidant, and antibacterial activities.^68^ Harnessing the interaction between phenolic compounds and proteins, Minari et al. developed physically cross-linked iongels formed by a gelatin–tannic acid polymer network and cholinium carboxylate ILs, which possessed high ionic conductivity values at room temperature (∼1.5 × 10^–2^ S cm^–1^), cell compatibility, and excellent performance in electrodes for electrophysiology.^69^ Nevertheless, although the iongels exhibited a thermo-reversible transition at 60 °C, this high-temperature injectability limited direct use as injectable iongels in the human body.

**Figure 4:**
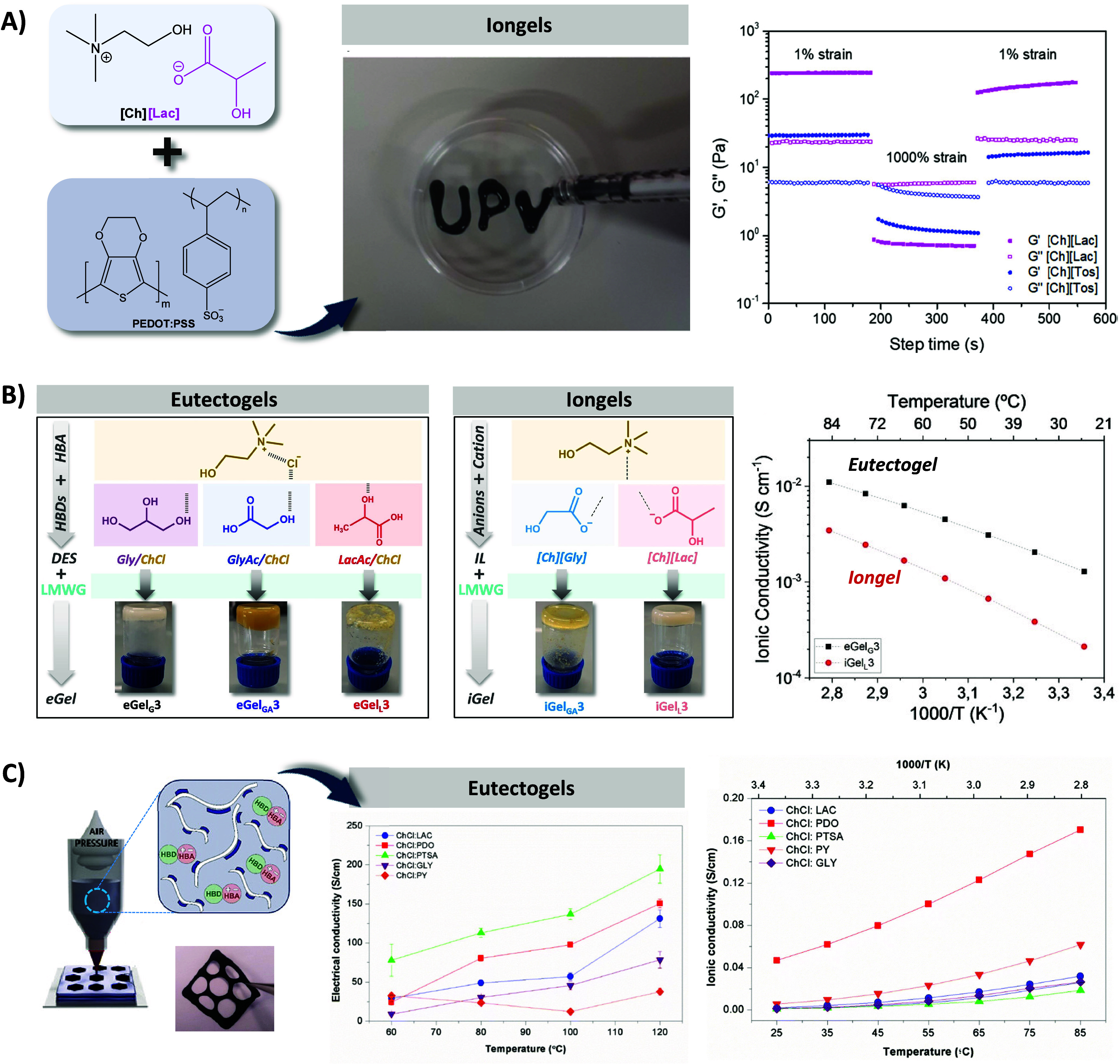
A) Chemical structures of PEDOT:PSS and ILs used to prepare injectable iongels. Picture of the injectability test of PEDOT:PSS/[Ch][Lac] and rheological dynamic step strain tests of the iongels PEDOT:PSS/[Ch][Lac] and PEDOT:PSS/[Ch][Tos]. Reproduced/adapted with permission from reference (12). Copyright 2022 The Royal Society of Chemistry. B) Eutectogels formed by the supramolecular interactions of different choline-chloride-based DES with a glutamic-acid-derived LMWG, with their corresponding inverted vial test pictures. Iongels formed by the supramolecular interactions of cholinium-based ionic liquids and the LMWG, with their corresponding inverted vial test pictures. Reproduced with permission from reference (13). Copyright 2023 Wiley-VCH GmbH. C) Schematic representation of the 3D extrusion printing used to fabricate shape-defined tattoo-like electrodes from eutectogels made of PEDOT:PSS and DES, and picture of the printed electrode with the PEDOT:PSS/ChCl:Lac eutectogel. Electrical and ionic conductivities of 50/50 wt % PEDOT:PSS/DES eutectogels. Reproduced/adapted with permission from reference (76). Copyright 2023 The Royal Society of Chemistry.

Intriguingly, an alternative approach involved incorporating ionic liquids (ILs) into conductive PEDOT:PSS aqueous dispersions. This strategy aimed to enhance electronic conductivity ultimately leading to the development of organic mixed ionic/electronic conductive materials (OMIECs).^70−72^ Following a similar approach, some of us recently showed injectable mixed ionic/electronic conductive gels made of PEDOT:PSS and biocompatible cholinium-type ionic liquids.^73^ The reversible electrostatic interaction between PEDOT and the IL that drove the iongel formation enabled the development of a series of injectable iongels under ambient conditions by systematically varying the cholinium derivative ionic liquid utilized in the process (Figure 4A). The rheological dynamic strain step test proved the iongels presented a reversible transition from a gel-like state at low strains to a liquid-like state at high strains, showcasing their inherent self-healing and injectability properties. Among the various ILs investigated, [Ch][Lac] possessed the highest electronic conductivity (∼30 S cm^–1^), which was 2 orders of magnitude higher than that of the naked PEDOT:PSS dispersion (∼0.2 S cm^–1^), while iongels formed with [Ch][Tos] possessed the highest ionic conductivity.^12^

### 2.3 Conductive Polymer Injectable Eutectogels or Gels Based on Deep Eutectic Solvents

In the last years, eutectogels have emerged as a new class of soft ionic conductive materials to be used as an alternative to conventional hydrogels and costly iongels to build wearable sensors and bioelectrodes.^30,74^ They consist of a cross-linked polymer network and a deep eutectic solvent (DES) that acts as a solvent and swelling agent. In a pioneering work, Picchio et al. developed mixed ionic/electronic conductive eutectogels by dispersing PEDOT:lignin sulfonate in a DES made of choline chloride/glycerol (ChCl/Gly), followed by the subsequent mixing with an aqueous gelatin solution at 90 °C. Physically cross-linked eutectogels were formed through H-bonding and charge stabilization upon cooling, enabling processing through 3D extrusion printing at 60 °C to obtain shape-defined eutectogels. As intended, the eutectogels showed high ionic (σ_i_ = 7.3 × 10^–3^ S cm^–1^) and electronic (σ_e_ = 8.7 × 10^–3^ S cm^–1^) conductivities.^75^

Very recently, we reported the first example of mixed ionic/electronic conductive eutectogel composites formed by the supramolecular self-assembly of a low-molecular-weight gelator (LMWG) combined with PEDOT: chondroitin sulfate (CS) and a glycine/choline chloride (Gly/ChCl) DES. Different DES, based on ChCl and three HBDs, namely glycerol (Gly = G), lactic acid (LacAc = L), and glycolic acid (GlyAc = GA), were tested (Figure 4B). Inverted vial tests show the eutectogel formation in the case of eGel_G_ and eGel_GA_, but not for eGel_L_, with *G*′ ranging from 350 and 1500 Pa. Besides, the comparison of eutectogels vs. iongels ([Ch][Lac] and [Ch][Gly]) was addressed. The eGel_G_3 eutectogels exhibited higher ionic conductivity (σ_i_ = 10^–2^ S cm^–1^) than iGel_L_3 iongels (σ_i_ = 10^–3^ S cm^–1^) due to the lower viscosity of Gly/ChCl DES compared with [Ch][Lac] IL, favoring the ionic mobility. These eutectogels also exhibited fluorescence, self-healing, and biocompatibility properties to be used as injectable materials for bioimaging.^13^ In a separate study, we delved into the impact of diverse DES on the formation of PEDOT:PSS/DES eutectogels at room temperature, along with their influence on ionic and electrical conductivity. We maintained a constant hydrogen bond acceptor (HBA)—choline chloride—while varying the hydrogen bond donor (HBD) used—OH-containing molecules such as glycerol (Gly) and 1,3-propanediol (PDO), natural organic acids like lactate (Lac), and aromatic acids including pyrogallol (PY) and *p*-toluenesulfonic acid (PTSA). Eutectogels produced from ChCl:PDO and ChCl:PY exhibited the highest ionic conductivity (1.6 × 10^–1^ and 4 × 10^–2^ S cm^–1^, respectively). Conversely, those featuring electron-rich DES architectures (ChCl:PY and ChCl:PTSA) displayed lower ionic conductivities (3 × 10^–2^ and 1 × 10^–2^ S cm^–1^, respectively) (Figure 4C). Our findings also revealed that the electronic conductivity mechanism prevailed in PEDOT:PSS/ChCl:PTSA, PEDOT:PSS/ChCl:PY, and PEDOT:PSS/ChCl:Lac eutectogels, while PEDOT:PSS/ChCl:PDO and PEDOT:PSS/ChCl:Gly eutectogels were dominated by ionic mechanisms. Just as previously demonstrated, the reversible physical cross-links enabled the additive manufacturing of these eutectogels through 3D extrusion printing at room temperature, facilitating the creation of tattoo-like electrodes for electrophysiology.^76^

## 3 Biomedical Applications of Conductive Injectable Gels

Bioelectronic medicine encompasses a broad range of applications targeting various diseases and disabilities, including cardiovascular, inflammatory, and neurodegenerative diseases, among others. The interface between biological materials and the electroactive gels plays a pivotal role in determining the success of the final biomedical application. Research efforts have predominantly concentrated on investigating the chemical, mechanical, biological, and functional properties of these interfaces to advance the field.^77,78^ The applications of electroactive injectable hydrogels, iongels, and eutectogels in bioelectronics primarily fall into two categories: tissue engineering and biosensors for electrophysiology.

### 3.1 Tissue Engineering

Electrical stimulation can activate intracellular signaling pathways in electro-responsive cells such as endothelial cells, cardiomyocytes, myoblasts, osteoblasts, and neural cells. This, in turn, influences cellular migration, proliferation, and differentiation, making electroactive injectable hydrogels valuable for tissue regeneration purposes.^79^ Electroactive injectable hydrogels have been used in a diverse range of applications from cell scaffolding, tissue culture, and wound repair up to on-tissue writing and 3D printing of wearable devices. The gelation process of physically and dynamic covalent cross-linked injectable hydrogels usually does not involve organic or electrochemical reactions, which facilitates the efficient encapsulation of electroresponsive cells within the gel network, enhancing the effectiveness of tissue engineering therapies.

For example, mesenchymal stromal cells (MSCs) were successfully encapsulated into PEG-peptide/PEDOT:PSS hydrogels, C2C12 myoblasts were laden into GelMA/PEDOT:PSS hydrogels for tissue regeneration therapies.^37,38^ Zhenan Bao and co-workers also developed injectable 3D cell-laden conductive hydrogels. They were formed by mixing an aqueous PEDOT:PSS solution with a concentrated solution of PBS. The ions in the PBS solution screened the electrostatic repulsions between the negatively charged PSS-rich colloidal suspensions, allowing them to aggregate into a solid-like gel (∼1 kPa). Then, the gels were immersed in a solution of acetic acid to induce transition to an electrically conductive morphology (∼10 S m^–1^). These injectable gels were further mixed with human induced pluripotent stem cell (iPSC)-derived neural progenitor cells (NPCs). The live/dead assay revealed the viability of NPCs encapsulated in the hydrogels on day 2. The biocompatibility of the gels was evidenced by the minimal inflammatory response when injected into a rodent brain.^80^

#### 3.1.1 Skin Wound Healing

The efficacy of injectable hydrogels in supporting cell growth and serving as templates for skin wound healing is contingent upon the integration of various key factors. CHI-CD-AD/GO hydrogels exhibited fast self-healing and electronic conductivity levels akin to skin, plus long-lasting antibacterial activity, hemocompatibility, and noncytotoxicity properties, as determined through *in vitro* coculturing red blood cells (RBC) and mouse fibroblasts (L929 cells). Furthermore, *in vivo* assessments revealed that these hydrogels facilitated an accelerated healing process, evidenced by heightened epidermis and granulation tissue thickness, increased collagen coverage area, and elevated expression of vascular endothelial growth factor (VEGF). In comparison to both the commercial Tegaderm dressing and the nonconductive CHI-CD-AD hydrogel, CHI-CD-AD/GO hydrogels demonstrated superior performance in the context of full-thickness skin repair.^58^

**Figure 5:**
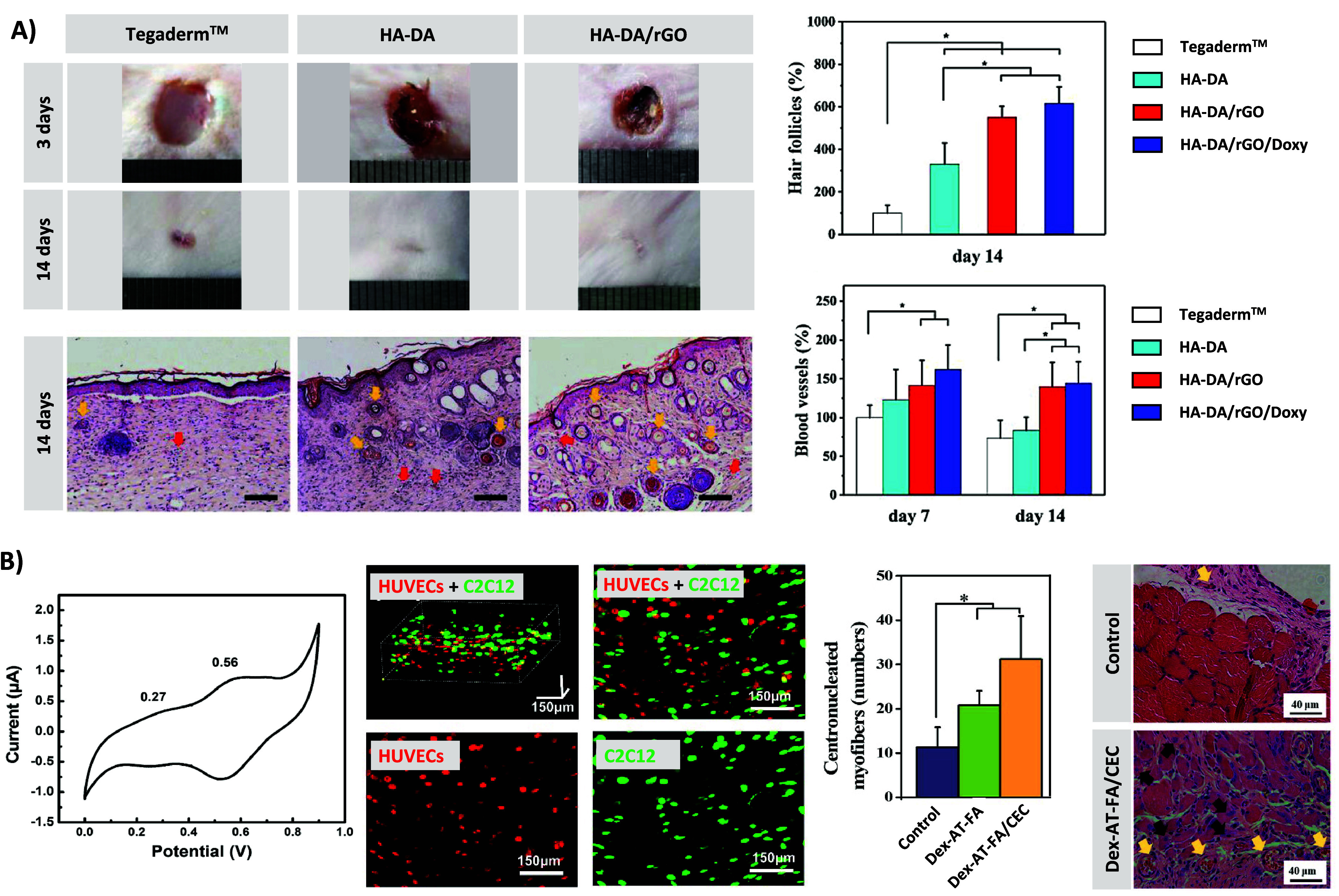
A) Photographs of wounds on days 3 and 14 (top-left) for Tegaderm film dressing (control), HA-DA, and HA-DA/rGO hydrogels. Pictures of histological analysis for wound regeneration on day 14 for Tegaderm (control), HA-DA, and HA-DA/rGO hydrogels (bottom-left). (blood vessels: red arrows, hair follicles: yellow arrows, boundary of epithelium: green lines). Scale bar = 100 μm. Hair follicles and blood vessels regeneration for different groups over 14 days (right). Reproduced/adapted with permission from reference (57). Copyright 2019 WILEY-VCH Verlag GmbH & Co. KGaA, Weinheim. B) CV curve of Dex-AT/CEC hydrogel. Confocal laser scanning microscopy images of C2C12 cells and HUVECs cocultured and injected in Dex-AT3/CECS hydrogels after 2 h. *In vivo* evaluation of skeletal muscle tissue regeneration in a rat model. Bar plot representing the newly formed centronucleated myofibers and representative H&E staining images of hydrogels and PBS control after implantation for 1 week. Reproduced/adapted with permission from reference (50). Copyright 2018 Elsevier.

In addition to this, adhesion properties are an added value to be taken into account. HA-DA/rGO hydrogels emerged as optimal choices for wound dressing due to their mechanical properties closely resembling human skin, coupled with noteworthy tissue adhesiveness. These hydrogels also exhibited antioxidant activity, fostering enhanced vascularization by upregulating the expression of the CD31 growth factor, which contributed to improving the granulation tissue thickness and collagen deposition. Interestingly, in a mouse full-thickness wound model, the HA-DA/rGO hydrogels outperformed both the commercial Tegaderm and nonelectroconductive HA-DA hydrogels in promoting superior wound closure (Figure 5A). Intriguingly, these hydrogels containing graphene oxide (GO) showcased the development of epithelial tissue, along with an increased presence of skin appendages such as hair follicles and blood vessels. This phenomenon was attributed to the conductivity of GO, potentially facilitating the transmission of electrical signals between wound sites and excitable skin cells, thereby promoting skin tissue regeneration.^57^

Similar properties were noted in the case of CHI-PANi/PEG-PGS and OHA-AT/CEC hydrogels as well.^46,48^ On the other hand, similar findings were also observed in hydrogels with ionic conductivity properties. Specifically, CMCHI-CMC-DA/NaCl hydrogels demonstrated injectability, as confirmed by successful extrusion through a syringe. These hydrogels were utilized to encapsulate human vein endothelial cells (HUVEC) and human dermal fibroblasts, revealing significant proliferation for both cell types after 5 days. Upon subcutaneous implantation in rats, the tissue architecture in the hydrogel-injected area was assessed using H&E staining. The biocompatibility was confirmed by the absence of inflammatory cells, and a normal tissue architecture was observed in the implanted site, indicating complete regeneration of the injured tissue. In contrast, H&E sections obtained from untreated control and CMCHI-CMC implanted sites exhibited distorted tissue architecture, suggesting incomplete regeneration of the injured tissue.^65^

The shear-thinning behavior and self-healing properties of injectable hydrogels, together with their noncytotoxic properties, made them accurate for on-skin printing and on-tissue writing bioelectronics. Alg-CA/PEDOT:PSS hydrogels were printed on human skin using syringe extrusion printing, resulting in the creation of on-skin printed electronics.^36^ Going one step further, Jin et al. studied the electrical performance of hydrogels comprising adhesive carboxymethyl cellulose, tannic acid, and metal ions (such as HAuCl_4_). These hydrogels were directly printed on skin tissue in diverse patterns, showcasing consistently stable conductive properties even when subjected to deformation through tension, bending, and twisting.^62^

#### 3.1.2 Muscle Tissue Engineering

Muscle tissues are composed of unidirectionally aligned myotubes forming skeletal muscle fibers; thus, the interaction between hydrogels and muscle cells plays a pivotal role in the advancement of injectable hydrogels for muscle tissue engineering.^81^ In this sense, PPy-MAC-Fe hydrogels were demonstrated to be noncytotoxic to C2C12 myoblast cells, with cells exhibiting a spindle-shaped morphology and high viability. Additionally, these hydrogels exhibited anticoagulant activity and showed blood compatibility.^45^ Electroactive scaffolds are recognized for their ability to autonomously provide electrical stimulation to cells in direct contact, facilitating chemical exchanges and signal communication with cells at the membrane–material interface. This phenomenon promotes cell alignment, as demonstrated in our recent work with 8220 muscle cells cultured on PEDOT-based printed scaffolds. Scaffolds made of PEDOT induced a distinct alignment of myoblasts and their subsequent differentiation into myotubes, following the specific strand direction of the printed pattern.^82^

Guo et al. tested the capacity of electroactive hydrogels made of Dex-AT-FA and CEC, which showed two oxidation transitions along with the increase in voltage, to encapsulate C2C12 myoblast cells and HUVEC endothelial cells within their network (Figure 5B). Results pointed out the uniform cell distribution of C2C12 cells and HUVECs when they were encapsulated together after 2 h. It was also observed that C2C12 cells were released from the hydrogels, being even able to proliferate after the release. In addition, these conductive Dex-AT-FA/CEC hydrogels were employed for the regeneration of the skeletal muscle in a volumetric muscle loss injury model. H&E staining images of regenerated skeletal muscle tissues after treatment with blank control and Dex-AT-FA/CEC hydrogel groups for 1 week showed that Dex-AT-FA/CEC hydrogel induced a higher myofiber density, more capillaries, and centronucleated myofibers in the muscle tissue defect than the control group.^50^

Zhang and co-workers investigated the encapsulation of C2C12 myoblasts into PEDOT:HEP/LM nanodroplets hydrogels, revealing notable outcomes. The hydrogels exhibited high cardiomyocytes cell viability with cell proliferation and formation of spheroid structures after 7 days. Under differentiation conditions, the myogenesis process was evaluated with the troponin T marker. After 3 days, there was a marked increase in troponin T expression for cells in the electroactive hydrogel compared to the nonconductive hydrogel. However, partial hydrogel degradation occurred due to polymer hydrolysis induced by enzymes secreted by the cells. In a subsequent experiment, these hydrogels were injected subcutaneously into immunocompetent hairless mice. After 25 days, histological analysis demonstrated that the materials did not elicit adverse inflammation, with no significant expression of inflammatory markers such as thrombomodulin and CD68.^41^

In addition to providing direct electrical stimulation to cells, Lu et al. explored the electroactive properties of PEDOT/sulfonated lignin/PAAm hydrogel. They assessed the *in vitro* proliferation and spreading of C2C12 cells on the hydrogels under various electrostimulation conditions (0, 300, and 600 mV). Conductive hydrogels demonstrated superior cell proliferation activity compared to nonconductive counterparts at 0 and 300 mV, although proliferation declined at the highest potential (600 mV). Moreover, cell spreading was enhanced in conductive hydrogels, with focal adhesion points exhibiting increased size when electrostimulation potential was below 300 mV.^31^

In a different study, *in vitro* bone marrow stem cell (BMSC) adhesion and proliferation were tested in contact with hybrid injectable DA-PAAm-GO hydrogels, in which the free catechol groups of polydopamine chains interacted with amine or thiol groups, forming cation−π or π–π interactions on the cell membrane. Under electrical stimulation, BMSC cell growth and proliferation were enhanced at 300 mV and 600 mV, exclusively in hydrogels containing GO. However, at higher voltages (900 mV), the cell growth rate decreased due to the harmful effects of high current, leading to cell death. When employed as an intramuscular hydrogel electrode, it exhibited dorsal muscle signals (0.1–40 mV) surpassing those detected by surface electrodes. The hydrogels had good long-term biocompatibility and tissue affinity *in vivo*. After implantation in a critical-sized osteochondral defect in a rabbit model, histological analysis revealed intimate contact of the hydrogel with surrounding tissue, accompanied by the formation of complete and mature cartilage tissue covering the defect area.^83^ In this vein, Choi et al. explored the application of cell-compatible Alg-BA/OEGCG/NaCl hydrogels for transmitting electromyography signals in an *ex vivo* muscle defect model. In this study, the hydrogel was positioned between parallel hind limb muscle tissues of a rat. The proximal muscle lump was then electrically stimulated (8 V amplitude pulse, 1 Hz), and the action potential signal in the distal muscle lump, transmitted through the conductive hydrogel, was recorded. Analysis of the electromyogram revealed no significant differences in the muscle–hydrogel–muscle signal (approximately 16 mV) when compared to the muscle-to-muscle direct contact model (approximately 17 mV), substantiating the hydrogel’s efficacy in transferring the action potential from the stimulated muscle tissue to the recorded tissue.^63^

#### 3.1.3 Cardiac Tissue Engineering

Acute Myocardial Infarction (MI) is one of the leading causes of mortality worldwide, primarily attributed to the absence of pharmacological treatments capable of reversing disease progression effectively. For those in advanced stages of heart failure, heart transplantation remains the sole viable option.^84^ Myocardial Infarction (MI) instigates cardiomyocyte demise, disrupts electrical communication, hampers biological exchanges through fibrotic scar formations, and induces a shortage of blood supply, ultimately leading to heart failure. Injectable hydrogels emerge as promising treatments for cardiac therapies following MI.

**Figure 6:**
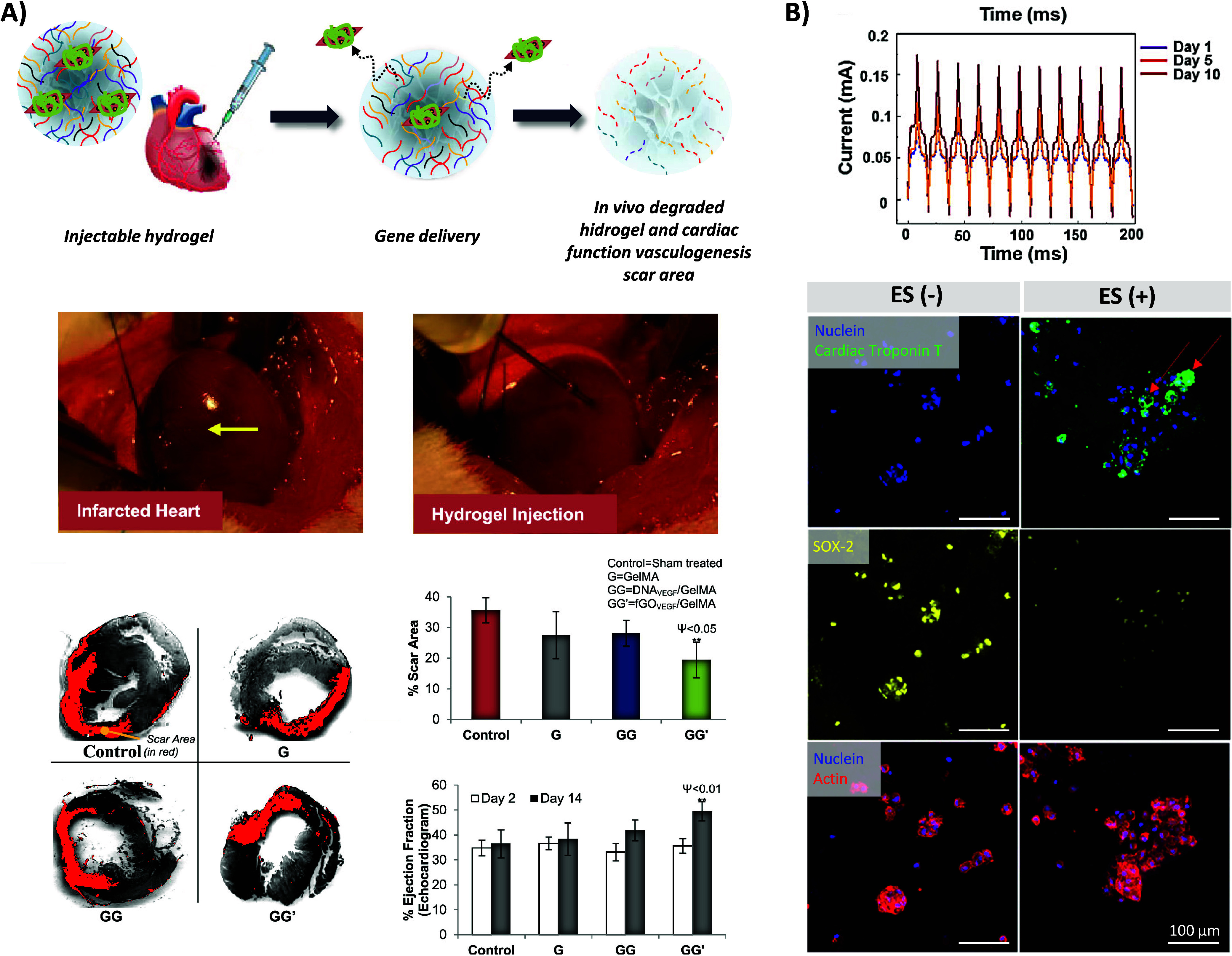
A) Schematic representation of the injection process of gene-loaded GO/GelMA hydrogels to treat damaged hearts with acute MI. Photographs of a myocardially infarcted rat heart (arrows indicate the damaged area in the left ventricular region) and direct intramyocardial delivery of injectable GO/GelMA hydrogel to the peri-infarct zones. Representative images of left ventricle myocardial sections stained with Sirius red to show the cardiac fibrosis regions (in red). Sham-operated and untreated infarcted groups were used as controls. The red area represents extracellular matrix (ECM) deposition in the scar tissue, and the gray area represents the myocardium. Echocardiographic assessment of cardiac function, in which heart ejection fraction (EF%) was monitored at days 2 and 14 post-treatment. Data are expressed as mean value ± SD. ANOVA statistical analysis with Bonferroni posthoc test was performed to determine the significance of the experiments. ****p < 0.0001; ***p < 0.001, **p < 0.01, *p < 0.05 vs time-matched control (*n* = 7). *p*-values comparing time-matched *GG*′ and *GG* are indicated by ψ. Reproduced/adapted with permission from reference (85). Copyright 2014 American Chemical Society. B) Pulse current of continuous ES at 1, 5, and 7 days. Cardiac troponin T (cTnT) and SOX-2 immunofluorescence staining of MSCs cultured on 0.5 mM PEG-peptide and 1% PEDOT:PSS hydrogels after 10 days. ES(−): without ES; ES(+): with ES; scale bars = 100 μm. Reproduced/adapted with permission from reference (37). Copyright 2018 American Chemical Society.

In one instance, biocompatible GO/GelMA nanocomposite hydrogels, incorporating vascular endothelial growth factor (VEGF), were designed in alignment with clinical needs. They were intramyocardially injected into the peri-infarct regions of a rat model with acute MI (Figure 6A), and results indicated that conductive hydrogels contributed to increased myocardial capillary density in the injected area, fostering local myocardial neovascularization. This led to a reduction in the scar area, an effect not observed with nonconductive hydrogels. Importantly, there were no significant differences in inflammatory markers at the injection sites, collectively improving cardiac function post-MI.^85^

Recently, Qian and co-workers developed biocompatible injectable hydrogels, formed by the Schiff base reaction between oxidized xanthan gum (OXG) and PPy grafted onto gelatin, OGGP3, which possessed good biocompatibility and mechanical and electrical properties that match those of heart tissue to reverse the adverse effects of MI and improve cardiac function. When injected into the rat myocardial scar tissue 2 days after MI, they found an enhanced cardiac function with a lower risk of arrhythmia in the rats treated with OGGP3, and a reduction of the electrical resistivity of myocardial fibrous tissue coupled with an increase of the conduction velocity of myocardial tissue. Four weeks after injection, a standard clinically programmed electrical stimulation (PES) protocol was applied to determine arrhythmia inducibility. Histological analysis of rats treated with OGGP3 revealed a decrease of the infarct size together with induced angiogenesis, thus showing its potential for the clinical treatment of MI.^86^

In another study, injectable CHI/COL/GQDs hydrogels were also tested in cardiac therapies post-MI. Initial assessments involved *in vitro* evaluations of human coronary artery endothelial cells (HCECs) cultured on the hydrogels. The conductive GQDs-loaded CHI/COL/GQDS hydrogels exhibited significantly enhanced cell viability and proliferation compared to nonconductive CHI/COL hydrogels after 18 h. Further investigations over 5 days revealed favorable outcomes in terms of cardiac progenitors and cardiomyocyte mitotic activity. This was evaluated using early cardiac transcription factors with cardiomyocyte progenitor (hCMPs) cells. Moreover, human mesenchymal stem cells (hMSCs) encapsulated within the hydrogels demonstrated heightened cell viability, increased expressions of pro-inflammatory and pro-angiogenic factors, and early cardiogenic markers. After injection of hMSCs-laden hydrogels into the intramyocardial area of an animal myocardial infarction model, echocardiography results after 30 days highlighted a significant reduction in left ventricle remodeling—a crucial factor in averting heart failure postmyocardial infarction. Moreover, there was a noticeable improvement in ejection fraction, reduced fibrosis area, increased vessel density, and diminished infarction size. These findings collectively underscore the potential of CHI/COL/GQDs hydrogels in enhancing heart regenerative function after MI, offering promising avenues for cardiac therapies.^56^

Another example reports the encapsulation of C2C12 myoblasts and H9c2 cardiac cells within CHI-PANi/PEG-PGS hydrogels. Interestingly, the release curves of C2C12 for 4 days and H9c2 cells for 7 days exhibited a linear-like cumulative cellular release pattern. This characteristic suggested that the hydrogels could offer a stable cell supply for cardiac tissue repair. Besides, *in vivo* studies involving the subcutaneous injection of the hydrogels into SD rats using a 22-gauge needle provided evidence of sustained cell retention and degradability, affirming the hydrogels’ potential for effective use in cardiac tissue repair.^49^ The native conductivity of the myocardium facilitates the transmission of impulse signals that play a crucial role in orchestrating heart contractions.^87^ Noncytotoxic scaffolds consisting of supramolecular CNTs/COL hydrogels were also engineered for cardiac tissue regeneration. The inclusion of carbon nanotubes (CNTs) in these scaffolds enhanced the alignment and anisotropic assembly of cardiomyocytes, particularly neonatal cardiomyocytes. This improvement resulted in a robust contraction potential, enabling the generation of synchronous contractions across the constructed tissues.^53^

In a recent work, Li and co-workers developed adipose-derived stem cells (ADSCs)-laden PEDOT:(Dex/CMCHI) hydrogels, which were tailored to match the mechanical properties and conductivity of the myocardium, serving as effective carriers for ADSCs delivery. The secreted factors, including hepatocyte growth factor (HGF), basic fibroblast growth factor (b-FGF), and vascular endothelial growth factor (VEGF) from the ADSCs, played a pivotal role in enhancing repair effects on damaged myocardium. These effects encompassed the inhibition of ventricular remodeling, reduction of fibrous scarring, promotion of vascular regeneration, and restoration of electrophysiological and pulsatile functions, which are key features to achieve better therapeutic outcomes and clinical translation.^41^

Recognizing the significance of adhesiveness and electrical stimulation in cardiac tissue regeneration, Xu et al. loaded mesenchymal stromal cells (MSCs) into PEG-peptide/PEDOT:PSS hydrogels. The inclusion of peptides significantly enhanced cell adhesion properties, leading to an increased number of spherical cell colonies observed after 5 days. Furthermore, the cell-laden hydrogels underwent electrical stimulation (ES) for 10 days, involving short pulses of 500 mV for 2 ms at intervals of 4 ms, administered for 8 h daily (Figure 6B). Over this period, the measured current gradually increased, aligning with progressive morphological changes in the cells, marked by the formation of nanofiber bundles around them. In addition, electro-stimulated cells exhibited an upregulation of the myocardiocyte marker cTnT after 10 days, while untreated cells remained cTnT-negative. Moreover, there was a reduced expression of the pluripotency marker SOX-2 in electro-stimulated cells, distinguishing them from untreated MSCs that developed into mesensphere-like structures. This indicates that the electro-stimulated cells underwent differentiation into myocardiocyte-like cells, underscoring the potential of this approach for cardiac tissue regeneration.^37^

#### 3.1.4 Neuronal Tissue Engineering

Nourbakhsh et al. devised a brain-compatible hydrogel meticulously tailored to replicate the electrical and mechanical properties of the hippocampus, a cerebral region crucial for learning and memory formation that is often susceptible to ischemic defects. Knowing that the vascular endothelial growth factor (VEGF) plays a key role in angiogenesis and neuronal surveillance following brain injuries, VEGF was loaded into electroconductive CHI-AP-Pluronic hydrogels. These VEGF-loaded hydrogels were subsequently injected into a rat model featuring ischemia-induced damage in the hippocampus. The study demonstrated a sustained and controlled release of VEGF over an extended period at the injury site. This resulted in a noteworthy 70% reduction in the infarcted size among rats treated with VEGF-hydrogel in comparison to the control group. Furthermore, this treatment exhibited enhanced hippocampal-dependent learning and memory performance, offering promising therapeutic potential for neurological disorders. The hydrogels showed complete degradation within 40 days under physiological conditions (PBS pH 7.4, 37 °C).^47^

### 3.2 Biosensors and Bioelectrodes

The use of biosensors to read and modulate the electrical activity within the body’s nervous system necessitates establishing a bidirectional interface between electrodes and biological systems to enable real-time diagnostics and therapeutics in bioelectronics medicine.^5,29,88^ Overall, the diverse capabilities of electroactive injectable gels, such as self-healing, stretchability, and conductivity, enable their utilization in 3D extrusion printing. This facilitates the construction of flexible wearable devices tailored for epidermal (e-skin) and subcutaneous biosensing applications.

#### 3.2.1 Biosensors Based on Injectable Hydrogels

Injectable PEDOT:PSS-based hydrogels were employed to fabricate soft neural probes, with impedances ranging from 50 to 150 kΩ at 1 kHz, for *in vivo* recording of neural activities. Upon implantation into the mouse dorsal hippocampus, it demonstrated the ability to successfully record continuous neural activities in a freely moving mouse over 2 weeks.^29^ In addition to neural probes, several strain sensors have also been developed.^59−61,89^ An illustrative example involved the utilization of PEDOT:PSS/CMC-DA hydrogels in crafting wearable, flexible, and long-term skin-adhesive strain sensors for human motion detection. These sensors effectively captured motion signals during finger or knee flexion at various angles (30°, 60°, and 90°), where resistance increased proportionally to the bending angle. When affixed to the throat, the sensors demonstrated the capability to detect resistance changes corresponding to vocal vibrations and speech-related motions, such as saying “hello” and “goodbye”.^35^

Strain sensors employing UPy/PANi/PSS hydrogels were created for tracking body movements such as finger and wrist bending, as well as subtle muscle activities including swallowing, speaking, and pulse beating. When positioned over the radial artery, the sensor exhibited the ability to detect blood pulse frequency, with the resistance change frequency reaching values of 80 beats/min under relaxed conditions and 115 beats/min after exercise.^51^ Furthermore, biocompatible electroactive PPy-MAC-Fe hydrogels, incorporating glucose oxidase (GOx), were utilized as injectable glucose sensors capable of detecting significant glucose levels within the range of 0–10 mM.^44^ In addition to this, mussel-inspired proanthocyanins (PC)-coated cellulose nanofibrils (CNF) were dispersed in a guar gum (GG) and glycerol solution to formulate PC-CNF-GG-glycerol hydrogels to create on-tissue portable noninvasive electrodes for detecting human electrophysiological signals, specifically for recording electrocardiograms (ECG) and electromyograms (EMG).^90^

#### 3.2.2 Biosensors Based on Injectable Iongels and Eutectogels

As previously highlighted, the interaction of hydrogels with ions present in biological fluids opens avenues for the development of ion-conducting gels, garnering increased attention in the creation of noninvasive electronics. Additionally, in the context of epidermal sensors, the potential water evaporation from hydrogels over their service life poses a risk of diminishing adhesive, mechanical, and electrical properties, thereby affecting their efficacy in bioelectronic applications. Consequently, recent endeavors have been dedicated to addressing this challenge through the utilization of iongels and, more recently, eutectogels. Injectable iongels with high ionic conductivity (1.8 × 10^–2^ S cm^–1^) and high-pressure sensitivity (0.1 kPa^–1^) were 3D printed and employed as pressure biosensors in contact with the fingers to detect human motions, as well as bioelectrodes for recording ECG showing comparable results to commercial medical electrodes.^67^

The integration of conducting polymers into iongels and eutectogels led to the creation of injectable mixed ionic/electronic conductive gels. Both iongels and eutectogels could be processed through 3D extrusion printing to fabricate customized biosensors. In this regard, injectable iongels, composed of choline lactate ([Ch][Lac]) and PEDOT:PSS, were used to produce flexible electrodes for EMG^73^ and ECG recordings.^12^ ECG results presented the characteristic waveform of heart activity (PQRST), which is crucial for cardiovascular disease diagnostics, and signal amplitudes and noise levels were comparable to those obtained with commercial electrodes (DENIS10026, Spes Medica). Furthermore, the stable signal baseline with low noise served as a hallmark of the excellent combined ionic (∼3 × 10^–6^ S cm^–1^) and electronic (∼30 S cm^–1^) conductivity of this composite electrode that made it well-suited for the fabrication of enduring wearable electronics.

**Figure 7:**
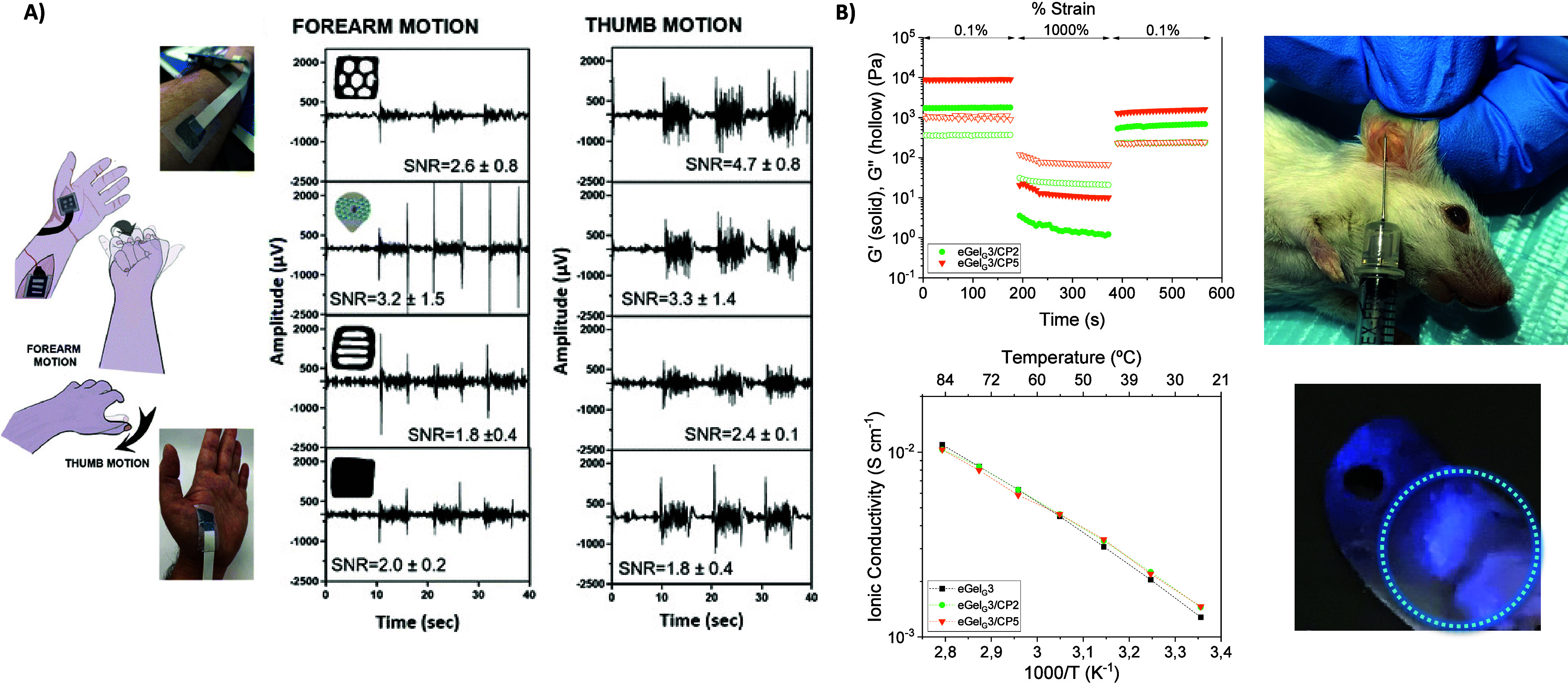
A) Schematic representation of the position of each eutectogel-based electrode placed on the skin and pictures of the tattoo-like electrodes in contact with the skin. EMG recordings on the forearm and thumb of hexagonal, striped, and solid tattoo-like electrodes, compared with Ag/AgCl electrodes. The signal-to-noise ratio (SNR) of each measurement is also shown. Reproduced/adapted with permission from reference (76). Copyright 2023 The Royal Society of Chemistry. B) Dynamic step-strain amplitude tests, and ionic conductivity of the injectable eutectogel composites, eGel_G_3/CP2 and eGel_G_3/CP5. *In vivo* injectability of the eutectogel composites in mice ears and the localized bioimaging fluorescence shown by the dashed blue circle. UV excitation at 254 nm. Reproduced/adapted with permission from reference (13). Copyright 2023 Wiley-VCH GmbH.

Additionally, in a recent development, injectable eutectogels based on choline chloride:lactic acid (ChCl/Lac) deep eutectic solvents (DES) and PEDOT:PSS were developed. These eutectogels were designed for use as long-lasting pressure biosensors^75^ and long-term bioelectrodes for health monitoring.^76^ Injectable PEDOT:PSS/DES eutectogels were able to be processed by 3D extrusion printing, leading to tattoo-like electrodes, which were placed *in vivo* on the forearm and the thumb of human volunteers for EMG measurements. In Figure 7A, the EMG signals recorded from the forearm exhibited a distinctive response similar to that of conventional Ag/AgCl electrodes across various printed geometries. Despite a lower signal-to-noise ratio (SNR) in eutectogels compared to commercial electrodes, there was a discernible trade-off between contact area and impedance values crucial for optimal recordings. When placed on the thumb, where electrode adaptability to the skin is essential, the hexagonally patterned eutectogels demonstrated superior performance (SNR = 4.7 ± 0.8) compared to commercial Ag/AgCl electrodes (SNR = 3.3 ± 1.4), stripped eutectogels (SNR = 2.4 ± 0.1), and solid eutectogels (SNR = 1.8 ± 0.4). Notably, the tattoo-like electrodes exhibited conformability to muscles, enabling measurements in intricate areas, resulting in improved recordings compared to commercially available electrodes. These electrodes demonstrated long-term stability and reusability, highlighting their potential for enhanced electromyographic applications.^76^

In addition to this, very recently, we pioneered the development of injectable mixed ionic/electronic eutectogels from a fluorescent glutamic-acid-derived low-molecular-weight gelator (LMWG) self-assembled in ChCl-based DES, with PEDOT:CS embedded within the network. Among different DES tested, supramolecular eutectogels formed with ChCl/glycerol DES showed the best self-healing and injectability properties. These dynamic materials displayed electronic (10^–4^ S cm^–1^) and high ionic (10^–2^ S cm^–1^) conductivities, as well as noncytotoxic properties in contact with induced pluripotent stem cells (iPSC). Furthermore, the eutectogels were successfully injected in subcutaneous local areas of rats exhibiting a localized fluorescence, which opened the way to potential minimally invasive bioimaging and electrostimulation applications (Figure 7B).^13^

## Conclusions and Outlook

This review article aimed to provide a comprehensive overview of the current developments and prospects in the emerging field of injectable polymer-based soft bioelectronics, a research topic that has garnered significant attention in the biomedical field in the past decade for diagnostic and healthcare treatments. A summary of the works referenced here is collected in Table 1, where the main types of injectable conductive gels are listed and related to their cross-linking mechanism, mechanical properties, conductivity, and biomedical applications. The research in electroactive polymer hydrogels involved the employment of conducting polymers such as PEDOT, PANi, and PPy, along with hybrid networks that comprised polymer hydrogels combined with conductive nanomaterials like carbon nanotubes, graphene oxide, and silver nanowires. Notably, PEDOT-based hydrogels took precedence in research, owing to their remarkable electronic conductivity, while PANi-based hydrogels garnered increasing attention due to their distinctive tissue adhesiveness features. From the injectability perspective, electroactive polymer networks cross-linked through physical interactions (e.g., electrostatic interactions, H-bonds, π–π stacking) or dynamic covalent bonds (e.g., imine bonds, Schiff base bonds, borate ester bonds) were chosen due to their remarkable shear-thinning and self-healing characteristics. These attributes made them ideal candidates as injectable bioelectronic materials for minimally invasive treatments focused on specific target areas.

**Table 1:** Main Types of Injectable Conductive Polymer-based Hydrogels, Iongels, and Eutectogels, Crosslinking Mechanism, Mechanical Properties, Conductivity, and Biomedical Applications

Type of gel^a^	Conducting material	Nonconductive material	Mechanical modulus^b^	Conductivity^c^	Cross-link	Application	ref
Hydrogel (I, P)	PEDOT:PSS	DMSO:H_2_O	*E* = 1.1 MPa	σ_e_ = 20 S cm^–1^	Physical	Biosensors	(29)
Hydrogel (I)	PEDOT:PSS	DBSA, PAAm, pHEMA	*E* = 1 to 10^2^ kPa	σ_e_ = 10^–1^ S cm^–1^	Physical interactions	C2C12 muscle cells growth	(34)
Hydrogel (I)	PEDOT:PSS	CMC-DA and PAAm	*G*′ ∼ 10^3^ Pa	σ_e_ = 41.6 S m^–1^	Dynamic covalent bonds	Biosensors	(35)
Hydrogel (I, P)	PEDOT:PSS	Alg-CA	*G*′ ∼ 10^2^ to 10^3^ Pa	σ_e_ = 2.5 × 10^–3^ to 4 × 10^–3^ S cm^–1^	Physical interactions	L929 mouse fibroblast cells growth	(36)
Hydrogel (I)	PEDOT:PSS	CWGG(BX)_n_ peptides	*G*′ ∼ 10^3^ Pa	σ_e_ = 35 Ω	Physical interactions	Cardiac regeneration	(37)
Hydrogel (I, P)	PEDOT:PSS	GelMA	*E* = 80 to 141.6 kPa	σ_e_ = 10^–5^ Ω	Physical and covalent interactions	C2C12 myoblasts cell-laden hydrogels	(38)
Hydrogel (I, P)	PEDOT:HEP	Dex-ALD-CH, CHI-CA, LM-TA	*G*′ ∼ 10^3^ Pa	σ_e_ = 5 × 10^–2^ S cm^–1^	Physical and dynamic covalent interactions	C2C12 myoblasts cell culture	(39)
						*In vivo* CT and MRI imaging	
Hydrogel (I)	PEDOT:Dex	CMCHI, ODex	*E* = 10–25 kPa	σ_e_ = 1.2 × 10^–2^ S cm^–1^	Dynamic covalent imine bonds	Cardiac regeneration	(41)
Hydrogel (I)	Tritriophene (ETE)	PVA, PLL	–	σ_e_ = 0.25 S cm^–1^	Physical interactions	*In vivo* bioelectrodes	(43)
Hydrogel (I)	PPy	MAC-Fe	*G*′ ∼ 1 to 3 kPa	σ_e_ = 3.4 × 10^–3^ S cm^–1^	Physical and covalent interactions	Glucose sensor	(44)
Hydrogel (I)	PPy	Gelatin-MAC-Fe	–	σ_e_ = 1.6 × 10^–2^ S cm^–1^	Physical interactions	Anticoagulant	(45)
Hydrogel (I)	PANi	CEC/OHA-AT	*G*′ ∼ 1 kPa	σ_e_ = 5 × 10^–5^ to 4.2 × 10^–4^ S cm^–1^	Schiff base bonds	Skin wound healing	(46)
Hydrogel (I)	PANi	CHI-NHS-AP-Pluronic	*G*′ ∼ 10 Pa	σ_e_ = 10^–3^ S cm^–1^	Schiff base bonds	Drug release	(47)
Hydrogel (I)	PANi	CHI and PEG-PGS	*G*′ ∼ 60 Pa to ∼ 370 Pa	σ_e_ = 3.5 × 10^–3^ S cm^–1^	Schiff base bonds	Skin wound healing	(48)
Hydrogel (I)	PANi	CHI-AT, PEG-DA	*G*′ ∼ 2 kPa to ∼12 kPa	σ_e_ = 2.5 × 10^–3^ S cm^–1^	Schiff base bonds	Cardiac repair	(49)
Hydrogel (I)	PANi	CEC and Dex-AT-FA	*G*′ ∼ 300 to 650 Pa	σ_e_ = 3.5 × 10^–5^ S cm^–1^	Schiff base bonds	Skeletal muscle repair	(50)
Hydrogel (I)	PANi	PSS-UPy	*G*′ ∼ 2 kPa to ∼ 6 kPa	σ_e_ = 1.3 × 10^–1^ S cm^–1^	Hydrogen bonds	Biosensors	(51)
Hydrogel (I)	GQDs	CHI/COL	–	–	Electrostatic interactions	Cardiac repair	(56)
Hydrogel (I)	rGO	HA-DA	*G*′ ∼ 10^2^ Pa	σ_e_ = 5 × 10^–3^ S cm^–1^	H-bonding	Skin wound healing	(57)
					π–π stacking		
Hydrogel (I)	GO	CHI-CD-AD	*G*′ ∼ 10^2^ Pa	σ_e_ = 1.1 × 10^–3^ S cm^–1^	Host–guest interaction	Skin wound healing	(58)
Hydrogel (I, P)	CNTs	PVA/HPC	*G*′ ∼ 10^2^ kPa	9.2 to 107 kΩ	Hydrogen bonds	Skin wound healing	(59)
Hydrogel (I)	Ag/MXene	PVA	–	σ_e_ = 3 × 10^–4^ to 2.6 × 10^–3^ S cm^–1^	Dynamic borate ester bonds and H-bonds	Biosensors	(60)
Hydrogel (I)	Ag NWs	Alg-PBA/Alg-DA	*E* = 15.6 to 22.6 kPa	σ_e_ = 2 × 10^–3^ S cm^–1^	Electrostatic interactions	Biosensors	(61)
Hydrogel (I, P)	HAuCl_4_, AgNO_3_, FeCl_3_	CMC/TA	*G*′ ∼ 1 kPa	3 to 12 kΩ	Hydrogen bonding and metal coordination	Biosensors	(62)
Hydrogel (I)	NaCl	Alg-BA/OEGCG	*G*′ ∼ 10^4^ Pa	1 kΩ	Boronic dynamic covalent bonds	Electromyography	(63)
Hydrogel (I)	NaCl, KCl	PAAm/CAR	*G*′ ∼ 10^4^ Pa	σ_i_ = 3.8 × 10^–2^ S cm^–1^	Hydrophobic, ionic interactions, and H-bonds	Biosensors	(64)
Hydrogel (I)	PBS	CMCHI/CC-DA	*G*′ ∼ 10^2^ Pa	σ_i_ = 3 × 10^–3^ S cm^–1^	Physical and dynamic covalent interactions	Tissue regeneration	(65)
Hydrogel (I)	PEDOT:PSS	PBS, acetic acid, HPMC	*G*′ ∼ 1 kPa	σ_e_ = 10 S m^–1^	Ionic interactions	iPSC cell-laden hydrogels	(80)
						*In vivo* biocompatibility	
Hydrogel (I)	GO	GelMA	*G*′ ∼ 10^2^ Pa	–	Physical interactions	Cardiac regeneration	(85)
Hydrogel (I)	PPy	OXG, gelatin	*G*′ ∼ 10^2^ Pa	σ_e_ = 5.5 × 10^–4^ S cm^–1^	Electrostatic interactions and Schiff base bonds	Cardiac regeneration	(86)
Hydrogel (I)	Glycerol, Na^+^ ions and Fe^3+^ ions	PC-CNF-GG	*G*′ ∼ 55 Pa	σ_i_ = 0.023 S cm^–1^	Ionic interactions	Biosensors	(90)
Iongel (I, P)	Cholinium-based ILs	PVA	*E* = 14 to 70 kPa	σ_i_ = 1.8 × 10^–2^ S cm^–1^	Physical interactions	Biosensors	(67)
Iongel (I)	[Ch][Sal], [Ch][Ga], [Ch][Van], [Ch][Caff]	PVA	*G*′ ∼ 10^2^ Pa	σ_i_ = 1.2 × 10^–2^ to 7.4 × 10^–4^ S cm^–1^	Physical interactions	Anti-inflammatory, antioxidant, antibacterial	(68)
Iongel (I)	Cholinium carboxylate ILs	Gelatin/TA	*E* = 11.3 to 28.9 kPa	σ_i_ = 1.5 × 10^–2^ S cm^–1^	Physical interactions	Biosensors	(69)
Iongel (I, P)	PEDOT:PSS, [Ch][Lac]	–	–	0.24 to 12 kΩ cm^–2^	Physical interactions	Biosensors	(73)
Iongel (I, P)	PEDOT:PSS, [Ch][Lac], [Ch][Tos]	–	*G*′ ∼ 10^2^ Pa	σ_e_ = 30 S cm^–1^	Physical interactions	Biosensors	(12)
				σ_i_ = 3 × 10^–6^ S cm^–1^			
Iongel (I)	PEDOT:CS,	LMWG	*G*′ ∼ 10^2^ Pa	σ_e_ = 10^–4^ S cm^–1^	Physical interactions	Bioimaging	(13)
Eutectogel (I)	[Ch][Lac], [Ch][Gly],		*G*′ ∼ 10^4^ Pa	σ_i_ = 10^–3^ S cm^–1^			
	Gly/ChCl, Lac/ChCl, GlyAc/ChCl			σ_i_ = 10^–2^ S cm^–1^			
Eutectogel (I, P)	PEDOT:ligning sulfonate, ChCl/Gly	Gelatin	*G*′ ∼ 10^4^ Pa	σ_e_ = 8.7 × 10^–3^ S cm^–1^	Physical interactions	Biosensors	(75)
				σ_i_ = 7.3 × 10^–3^ S cm^–1^			
Eutectogel (I, P)	PEDOT:PSS, ChCl/PDO, ChCl/PY, ChCl/PTSA, ChCl/Lac, ChCl/Gly	–	*G*′ ∼ 10^2^ Pa	σ_e_ = 10^1^ to 10^2^ S cm^–1^	Physical interactions	Biosensors	(76)
				σ_i_ = 10^–1^ S cm^–1^ to 10^–2^ S cm^–1^			

a (I) injectable, (P) printable.

b *E* and *G*′ stand for Young’s modulus and Elastic modulus, respectively.

c σ_e_ and σ_i_ stand for electronic and ionic conductivities, respectively.

These soft polymer-based hydrogels offered tunable mechanical and electrical properties showing mixed ionic electronic conducting behavior, allowing them to mimic the features of various body tissues. This versatility provided a wide range of bioelectronics materials for diverse biomedical applications, overcoming the limitations of traditional organic/inorganic electronics. However, hydrogels faced challenges such as water evaporation over their lifetime, leading to a potential loss of mechanical and electrical properties. To address this, iongels and eutectogels emerged as promising candidates in the polymer-based soft bioelectronics field, opening new research avenues for exploration in the coming years.

Furthermore, the ability of injectable mixed ionic/electronic conductive polymer gels (iongels and eutectogels) to be processed by 3D printing enabled the creation of flexible, wearable, shape-defined scaffolds and tattoo-like materials. The ability of customized injectable iongels and eutectogels to adhere and adapt to human skin (e-skin) and other body tissues paves the way for the development of the next generation of bioelectronic devices. Finally, it is worthy to remark that most studies were focused on the electrical stimulation provided by the gels to cells and tissues in contact with them, whereas there is growing interest in exploring the effects of external electrical stimulation during *in vivo* applications, presenting exciting prospects for future research.
